# Supplementary material for: Nuclear phase retrieval spectroscopy using resonant x-ray scattering
Source: Nat Commun. 2025 Mar 31;16:3096. doi: 10.1038/s41467-025-58396-z (PMC11958670; doi:10.1038/s41467-025-58396-z)
Supplement: Supplementary file 1 — Supplementary Information [file 41467_2025_58396_MOESM1_ESM.pdf]

Nuclear phase retrieval spectroscopy using resonant x-ray scattering
Supplementary Methods

THEORETICAL ANALYSIS FOR THE MODEL

A. Error analysis for the discrete model

We recall that the photon counts $\{I(t,\Delta_D)\}_{t\in\mathbf{E},\Delta_D\in\mathbf{\Omega}}$ are measured at a series of discrete time points $\mathbf{E}=\{t_1,t_2,\cdots,t_K\}$, where $\mathbf{\Omega}=\{\Delta_D^1,\Delta_D^2,\cdots,\Delta_D^L\}$. For each Δ_D, the corresponding analyzer transmission function $T(\Delta-\Delta_D)$ is known in advance. The continuous model

$$I(t,\Delta_D)=\left|\frac{1}{\sqrt{2\pi}}\int_{-\infty}^{+\infty}R(\Delta)T(\Delta-\Delta_D)e^{-i\Delta t}d\Delta\right|^2 \tag{1}$$

can be discretized to

$$I(t_k,\Delta_D^l)=\left|\mathbf{F}_{:,k}^{\mathrm{H}}(\mathbf{R}\odot\mathbf{T}_l)\right|^2+\varepsilon, t_k\in\mathbf{E},\Delta_D^l\in\mathbf{\Omega}, \tag{2}$$

with $\mathbf{F}_{:,k}=\frac{\Delta_{\text{step}}}{\sqrt{2\pi}}(e^{i0\Delta_{\text{step}}\cdot t_k},e^{i1\Delta_{\text{step}}\cdot t_k},\cdots,e^{i(n-1)\Delta_{\text{step}}\cdot t_k})$. The error ε in (2) comes from two sources: aliasing and truncation.

The aliasing is caused by sampling continuous variables. Note that any discrete signal can be represented as a sum of Dirac delta functions. As a result, a uniform sampling of $R(\Delta)T(\Delta-\Delta_D)$ with step Δ_{step} thus corresponds to the weighted sum of delta functions below

$$R_d(\Delta)T_d(\Delta-\Delta_D):=\sum_{h=-\infty}^{+\infty}R(\Delta)T(\Delta-\Delta_D)\delta(\Delta-h\Delta_{\text{step}}). \tag{3}$$

Next, we define

$$I_d(t,\Delta_D)\ :=\left|\frac{\Delta_{\text{step}}}{\sqrt{2\pi}}\sum_{h=-\infty}^{+\infty}R(h\Delta_{\text{step}})T(h\Delta_{\text{step}}-\Delta_D)e^{-ih\Delta_{\text{step}}t}\right|^2.$$

Notice that

$$\int_{-\infty}^{+\infty} \delta(\Delta - h\Delta_{\text{step}}) e^{-i\Delta t} d\Delta = e^{-ih\Delta_{\text{step}} t} .$$

By using (3), we have

$$\begin{aligned}
I_d(t, \Delta_D) &= \left| \frac{\Delta_{\text{step}}}{\sqrt{2\pi}} \sum_{h=-\infty}^{+\infty} R(h\Delta_{\text{step}}) T(h\Delta_{\text{step}} - \Delta_D) \int_{-\infty}^{+\infty} \delta(\Delta - h\Delta_{\text{step}}) e^{-i\Delta t} d\Delta \right|^2 \\
&= \left| \frac{\Delta_{\text{step}}}{\sqrt{2\pi}} \int_{-\infty}^{+\infty} \sum_{h=-\infty}^{+\infty} R(h\Delta_{\text{step}}) T(h\Delta_{\text{step}} - \Delta_D) \delta(\Delta - h\Delta_{\text{step}}) e^{-i\Delta t} d\Delta \right|^2 \\
&= \left| \frac{\Delta_{\text{step}}}{\sqrt{2\pi}} \int_{-\infty}^{+\infty} \sum_{h=-\infty}^{+\infty} R(\Delta) T(\Delta - \Delta_D) \delta(\Delta - h\Delta_{\text{step}}) e^{-i\Delta t} d\Delta \right|^2 \\
&= \left| \frac{\Delta_{\text{step}}}{\sqrt{2\pi}} \int_{-\infty}^{+\infty} R_d(\Delta) T_d(\Delta - \Delta_D) e^{-i\Delta t} d\Delta \right|^2 ,
\end{aligned}$$

which is the intensity of the Fourier transform of $R_d(\Delta)T_d(\Delta - \Delta_D)$.

To further study the effect caused by discretization, we note that the relationship between $I_d(t, \Delta_D)$ and $I(t, \Delta_D)$ reads

$$\begin{aligned}
I_d(t, \Delta_D) &= \left| \frac{\Delta_{\text{step}}}{\sqrt{2\pi}} \int_{-\infty}^{+\infty} R_d(\Delta) T_d(\Delta - \Delta_D) e^{-i\Delta t} d\Delta \right|^2 \\
&= \left| \frac{\Delta_{\text{step}}}{\sqrt{2\pi}} \int_{-\infty}^{+\infty} \sum_{h=-\infty}^{+\infty} R(\Delta) T(\Delta - \Delta_D) \delta(\Delta - h\Delta_{\text{step}}) e^{-i\Delta t} d\Delta \right|^2 \\
&\overset{(1)}{=} \left| \frac{1}{\sqrt{2\pi}} \sum_{p=-\infty}^{+\infty} \int_{-\infty}^{+\infty} R(\Delta) T(\Delta - \Delta_D) e^{-i\Delta(t - \frac{p}{\Delta_{\text{step}}})} d\Delta \right|^2 \\
&= \left| \overbrace{\frac{1}{\sqrt{2\pi}} \int_{-\infty}^{+\infty} R(\Delta) T(\Delta - \Delta_D) e^{-i\Delta t} d\Delta}^{(a)} \right. \\
&\quad \left. + \overbrace{\frac{1}{\sqrt{2\pi}} \sum_{p \neq 0} \int_{-\infty}^{+\infty} R(\Delta) T(\Delta - \Delta_D) e^{-i\Delta(t - \frac{p}{\Delta_{\text{step}}})} d\Delta}^{(b)} \right|^2 ,
\end{aligned} \tag{4}$$

where the step labeled with (1) in the above equation is deduced using the equality

$$\sum_{h=-\infty}^{+\infty} \delta\left(\Delta - h\Delta_{\text{step}}\right) = \frac{1}{\Delta_{\text{step}}} \sum_{p=-\infty}^{+\infty} e^{i\Delta \frac{p}{\Delta_{\text{step}}}},$$

and p and h are both integers. When Δ_D is given, (4) shows that $I_d(t, \Delta_D)$ is a $\frac{1}{\Delta_{\text{step}}}$ periodical signal which is equal to the intensity of all the summations of (a) shifted by $\frac{p}{\Delta_{\text{step}}}$. Thus, (b) is the source of aliasing when discretizing $R(\Delta)$ by a uniform step Δ_{step}. If $R(\Delta)T(\Delta - \Delta_D)$ is a band-limited signal with a cutoff time t_{c}, then the influence made by the term (b) can be reduced when $\Delta_{\text{step}} \leq \frac{1}{t_{\text{c}}}$. In addition to the induced error by (b), the energy resolution of the recovered spectrum and the computational complexity are also taken into account and $\Delta_{\text{step}} = 1\Gamma_0$ is adopted in our calculation.

Next, the error caused by truncation is attributed to the limitation of digital devices, where Δ is in the finite range $[-\Delta_{\max}, \Delta_{\max}]$. We divide $I_d(t, \Delta_D)$ into the addition of two parts below

$$\begin{aligned} &\left| \overbrace{\frac{\Delta_{\text{step}}}{\sqrt{2\pi}} \sum_{h=0}^{n-1} R(-\Delta_{\max} + h\Delta_{\text{step}}) T(-\Delta_{\max} + h\Delta_{\text{step}} - \Delta_D) e^{-ih\Delta_{\text{step}} t}}^{(c)} \right. \\ &\left. + \overbrace{\frac{\Delta_{\text{step}}}{\sqrt{2\pi}} \sum_{h \in (-\infty, 0) \cup [n, +\infty)} R(-\Delta_{\max} + h\Delta_{\text{step}}) T(-\Delta_{\max} + h\Delta_{\text{step}} - \Delta_D) e^{-ih\Delta_{\text{step}} t}}^{(d)} \right|^2, \end{aligned} \tag{5}$$

where (d) is usually truncated. When Δ_{step} is given, increasing $\Delta_{\max}$ can reduce the truncated error. But $\Delta_{\max}$ cannot be arbitrarily large, since it must satisfy $\Delta_{\max} = \frac{\pi}{t_{\text{step}}}$ with t_{step} being the smallest sampling interval. Most importantly, when the influence caused by (b) and (d) can be controlled, specifically once t_{step} is small and $\Delta_{\text{step}} \leq \frac{1}{t_c}$, then $I(t_k, \Delta_D^l) \approx \left|\mathbf{F}_{:,k}^{\text{H}}(\mathbf{R} \odot \mathbf{T}_l)\right|^2$ holds with high accuracy.

B. Solution analysis for the optimization model

In this Section, we discuss the solution of the following optimization problem: infer $\mathbf{R}$ from the given set of counts $\mathbf{I}$, i.e., find $\mathbf{R} \in \mathbb{C}^n$ such that

$$\begin{aligned} I(k,l) &= \left|\mathbf{F}^{\mathrm{H}}_{:,k}(\mathbf{R} \odot \mathbf{T}_l)\right|^2 + \varepsilon, \\ k &\in \{1,2,\cdots,K\}, l \in \{1,2,\cdots,L\}. \end{aligned} \tag{6}$$

Here, n is the size of $\mathbf{R}$. This is the discretized model introduced in Eq. (2) in the main manuscript. In general, the solution of (6) is not unique. One reason is that the global phase of $\mathbf{R}$ cannot be determined. For instance, if $\mathbf{R}_0$ is a solution of (6), then $\mathbf{R}_0$ with a global phase $\theta \in [0, 2\pi)$, $\mathbf{R}_0 e^{i\theta}$ also satisfies the constraints in (6). Note that the global phase θ is not under consideration in the experiment. Thus, when it comes to the unique solution of (6), we treat the solutions differing by a global phase as the same.

In Refs. [1–3], theoretical results proved that the solution of (6) can be unique up to the global phase under some suitable assumptions. For example, if the number of sampling points $K \geq n$, and the union of the windows W_l of $|\mathbf{T}_l|^2, l = 1, \cdots, L$ covers $[-\Delta_{\max}, \Delta_{\max}]$. However, these assumptions usually fail in practice. For instance, in order to compensate the error ε caused by discretization, we must choose Δ_{step} and t_{step} properly. This usually leads to K being less than n. At the same time, $\overset{K}{\underset{l=1}{\cup}} W_l$ usually cannot cover $[-\Delta_{\max}, \Delta_{\max}]$. Thus, recovering $\mathbf{R}$ uniquely from (6) is nearly impossible based on these under-determined conditions. Fortunately, in practice what we are concerned with is a fraction of $\mathbf{R}$ which is covered by $\overset{K}{\underset{l=1}{\cup}} W_l$. For example, the region of interest $\mathbf{O}$ in $\mathbf{R}$ is $[-100\Gamma_0, 100\Gamma_0]$ and $\overset{K}{\underset{l=1}{\cup}} W_l$ covers $[-170\Gamma_0, 170\Gamma_0]$ in the main text. This may guarantee $\mathbf{R}$ to be unique in the region of interest $\mathbf{O}$.

Next, numerical simulations are applied to verify the assumption that $\mathbf{R}$ is unique in the region $\mathbf{O}$. We choose the numerical results presented in the Fig. 2 of main text as the signal of interest. We consider three different sets of Doppler detunings, $\mathbf{\Omega} = -50\Gamma_0 : 50\Gamma_0$, $\mathbf{\Omega} = -100\Gamma_0 : 100\Gamma_0$, and $\mathbf{\Omega} = -150\Gamma_0 : 150\Gamma_0$. The other parameters are the same as the ones used for the simulations in Fig. 2 of main text. We generate $\mathbf{I}$ according to different $\mathbf{\Omega}$, and use the NPRS algorithm to recover $\mathbf{R}$ from the corresponding $\mathbf{I}$ respectively. The results are shown in Fig. 1.

Figures 1**a-d,f,g,i,j** show that the $\hat{\mathbf{R}}$ recovered by the NPRS algorithm fits the ground

truth $\mathbf{R}$ well in the range of $\mathbf{\Omega}$. However, the spectral intensity of $\hat{\mathbf{R}}$ cannot fit $\mathbf{R}$ well in those regions out of $\mathbf{\Omega}$. At the same time, the measurement error at each case is around 10^{-5}. This is absolutely sufficient for our purposes. We conclude that in order to recover $\mathbf{R}$ well within the the region $\mathbf{O}$, the range of the Doppler detuning set $\mathbf{\Omega}$ must cover $\mathbf{O}$.

INTRODUCTION TO THE NPRS ALGORITHM

The NPRS algorithm is used to solve the optimization problem

$$\underset{\mathbf{R}\in\mathbb{C}^n}{\text{minimize}}\, \ell(\mathbf{R}) = \sum_{k=1}^{K}\sum_{l=1}^{L}\left(\left|\mathbf{F}^{\mathrm{H}}_{:,k}(\mathbf{R}\odot\mathbf{T}_l)\right|^2 - I(k,l)\log\left(\left|\mathbf{F}^{\mathrm{H}}_{:,k}(\mathbf{R}\odot\mathbf{T}_l)\right|^2\right)\right). \qquad (7)$$

In the following, we present details of the NPRS algorithm, beginning with its formal steps presented in Algorithm 1.

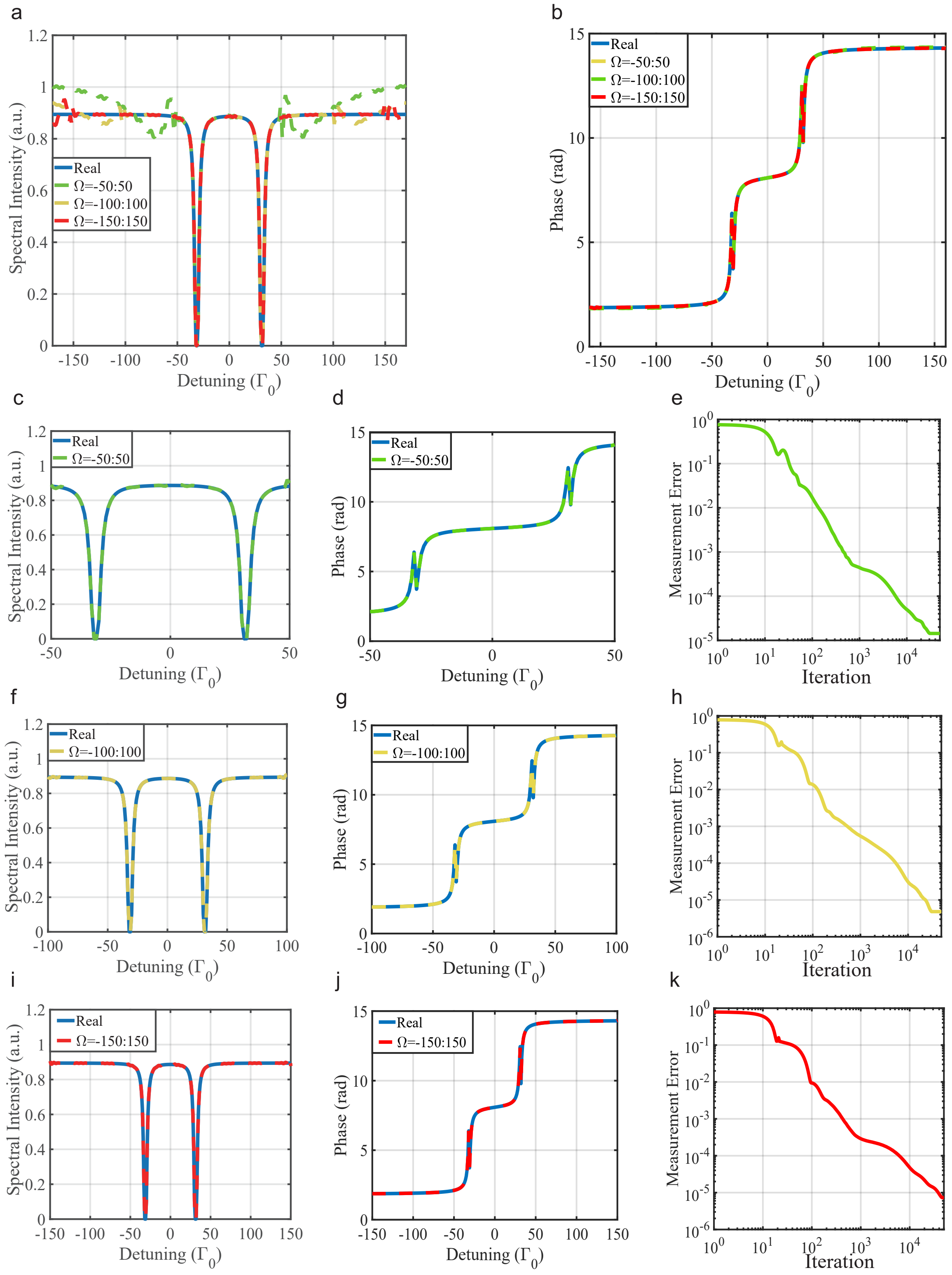

SUPPLEMENTARY FIG. 1. **Optimization of the Doppler detuning range.** **a**, **b** Energy spectrum and phase recovered by the NPRS algorithm using three choices of $\boldsymbol{\Omega}$. **c-d** NPRS spectrum and recovered phase for $\boldsymbol{\Omega} = -50\Gamma_0 : 50\Gamma_0$. **e** Measurement error at each iteration. **f-h** and **i-k** are the results for $\boldsymbol{\Omega} = -100\Gamma_0 : 100\Gamma_0$ and $\boldsymbol{\Omega} = -150\Gamma_0 : 150\Gamma_0$, respectively.

Algorithm 1: Nuclear Phase Retrieval Spectroscopy (NPRS)

Input:

$\mathbf{\Omega}$: the set of shifting detuning $\mathbf{\Omega} = \{\Delta_D^1, \Delta_D^2, \cdots, \Delta_D^L\}$

$\mathbf{E}$: the set of discrete time points $\mathbf{E} = \{t_1, t_2, \cdots, t_K\}$

$\mathbf{T}_l \in \mathbb{C}^n, l = 1, \cdots, L$: the analyzer with L different Doppler detunings

$\mathbf{I} \in \mathbb{R}^{K\times L}$: the counts of photons

μ: the step size

τ: the error bound

Output:

$\hat{\mathbf{R}}$: an estimation of $\mathbf{R}$

Initialization:

$\mathbf{R}^{(0)} \in \mathbb{C}^n$

$\tilde{\mathbf{R}}^{(0)} = \mathbf{R}^{(0)}$

$t = 1$

General Step $(m = 1, 2, \cdots)$:

$\tilde{\mathbf{R}}^{(m)} = \mathbf{R}^{(m-1)} - \mu\nabla\ell_\omega\left(\mathbf{R}^{(m-1)}\right)$

$\mathbf{R}^{(m)} = \tilde{\mathbf{R}}^{(m-1)} + \frac{t-1}{t+2}\left(\tilde{\mathbf{R}}^{(m)} - \tilde{\mathbf{R}}^{(m-1)}\right)$

if $\ell_\omega(\mathbf{R}^{(m)}) < \ell_\omega(\mathbf{R}^{(m-1)})$ **then**

$t = t + 1$

else

$t = 1$

end if

if $\ell_\omega(\mathbf{R}^{(m)}) \le \tau$ **then**

$\hat{\mathbf{R}} = \mathbf{R}^{(m)}$

Break

end if

C. Initialization

As stated in the main text, the optimization problem (7) is non-convex and its numerical method may be sensitive to the algorithm initialization. In this subsection, simulations will be applied to test the performance of the NPRS algorithm by using two different types

of initialization. For type I we use $\alpha\mathbf{1}$, where α is randomly chosen from $(0,1]$ as the initialization and $\mathbf{1}$ is a length n vector with each entry equal to 1. For type II, each entry of the initialization is sampled from the Gaussian distribution $\mathcal{N}(0,0.1)$. As signal of interest, we choose the nuclear forward scattering setup with aligned magnetization discussed in the main text for which numerical simulations are performed. This case is discussed in more detail later in Section G. The time range is chosen from 3 ns to 165 ns. Furthermore, $\Delta_{\text{step}} = 1\Gamma_0$, $t_{\text{step}} = 0.1974$ ns, $\mathbf{\Omega} = -150\Gamma_0 : 150\Gamma_0$, and $\mathbf{O} = -100\Gamma_0 : 100\Gamma_0$, where ':' denotes the interval range. The total iteration number is 2000. For each initialization, we randomly generate 25 samples and record their measurement errors and relative errors respectively. We recall here the definitions of the relative error [Eq. (4) in the main text]

$$\sqrt{\frac{\sum_{\Delta\in\mathbf{O}}\left|\hat{\mathbf{R}}(\Delta)e^{i\theta}-\mathbf{R}(\Delta)\right|^2}{\sum_{\Delta\in\mathbf{O}}\left|\mathbf{R}(\Delta)\right|^2}},$$

where $\theta \in \arg\min_{\theta\in(0,2\pi]}\left|\hat{\mathbf{R}}(\Delta)e^{i\theta}-\mathbf{R}(\Delta)\right|^2$, and the the measurement error [Eq. (5) in the main text]

$$\sqrt{\frac{\sum_{k=1}^{K}\sum_{l=1}^{L}\left(I(k,l)-\left|\mathbf{F}_{:,k}^{\mathrm{H}}(\hat{\mathbf{R}}\odot\mathbf{T}_l)\right|^2\right)^2}{\sum_{k=1}^{K}\sum_{l=1}^{L}I^2(k,l)}}.$$

The results are shown in Fig. 2. We find that the NPRS algorithm is robust with respect to initialization and converges quickly for both cases, regardless of the chosen initialization. At the same time, the results in Fig. 2 also show that the type I initialization can make the NPRS algorithm converge faster. We therefore choose for the following tests the type I initialization.

Compared with the vanilla gradient descent algorithm mentioned in Methods in the main text, the NPRS algorithm introduces two modules to make the gradient descent procedure more robust and converging more quickly: weighted loss and acceleration and restart. These are discussed in the subsections below.

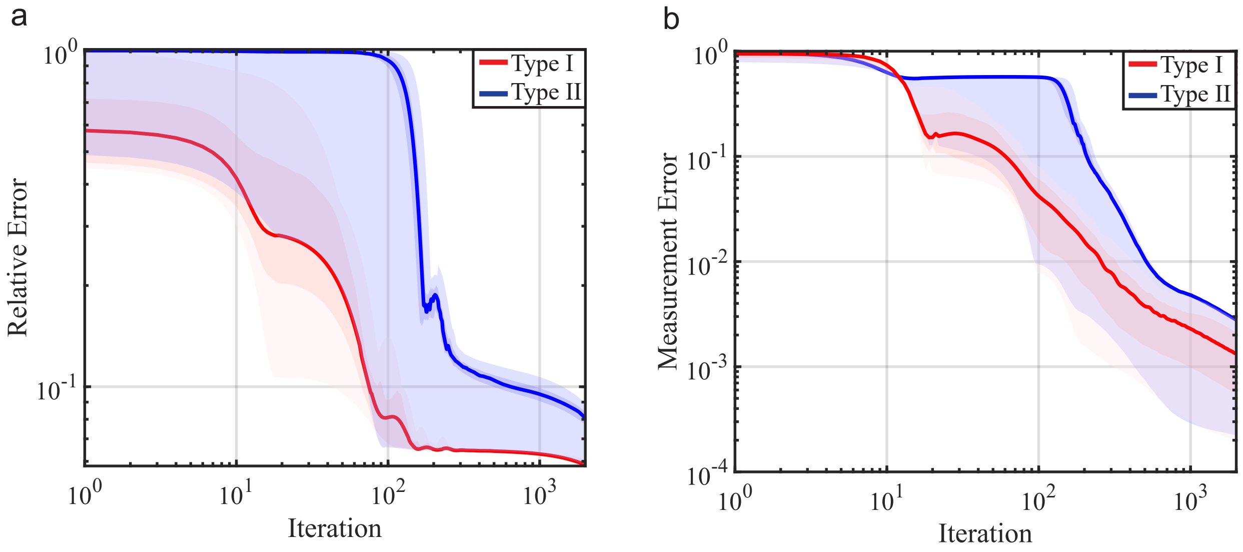

SUPPLEMENTARY FIG. 2. **Choice of initialization.** **a** Relative and **b** measurement errors of the retrieved response function as a function of algorithm iteration for two different types of initialization. The input 2D data set was generated by a numerical simulation of a nuclear forward scattering setup with aligned magnetization. The thick line shows the median over 25 trials. The light area highlights the entire region between minimum and maximum, and the darker area the region between the 25th and 75th percentile, respectively.

D. Weighted loss

Based on (7), we introduce a weighed loss alternative to prevent over-fitting:

$$\underset{\mathbf{R}\in\mathbb{C}^n}{\text{minimize}}\, \ell_\omega(\mathbf{R}) = \sum_{k=1}^{K}\sum_{l=1}^{L}\omega(k,l)\left(\left|\mathbf{F}^{\mathrm{H}}_{:,k}(\mathbf{R}\odot\mathbf{T}_l)\right|^2 - I(k,l)\log\left(\left|\mathbf{F}^{\mathrm{H}}_{:,k}(\mathbf{R}\odot\mathbf{T}_l)\right|^2\right)\right), \qquad (8)$$

where $\omega(k,l)=\omega_1(k,l)\omega_2(l)$ is the non-negative weight function which considers different effects on the loss depending on (k,l). The reasoning behind the formulation of $\omega(k,l)$ is twofold, as explained below.

First, $\omega_2(l)$ is used to balance the loss between different l values, for which we choose the Gaussian function

$$\omega_2(l) = e^{-\frac{(\Delta_D^l - u)^2}{2\sigma^2}}$$

with $u = \frac{1}{|\boldsymbol{\Omega}|}\sum_{\Delta_D^l\in\boldsymbol{\Omega}}\Delta_D^l$ typically in the vicinity of zero, and $\sigma^2 = \frac{1}{|\boldsymbol{\Omega}|}\sum_{\Delta_D^l\in\boldsymbol{\Omega}}(\Delta_D^l - u)^2$. In many tests we find that the error $\sum_{k=1}^{K}(I(k,l) - |\mathbf{F}^{\mathrm{H}}_{:,k}(\mathbf{R}\odot\mathbf{T}_l)|^2)^2$ has a large spread depending on the value of l during the iteration. Concretely, the error is small for large $|\Delta_D^l|$

and becomes larger for small $|\Delta_D^l|$. Thus, to prevent over-fitting so that $\left|\mathbf{F}_{:,k}^{\mathrm{H}}(\mathbf{R} \odot \mathbf{T}_l)\right|^2$ can fit $I(k,l)$ better when $|\Delta_D^l|$ is small, we use the Gaussian $\omega_2(l)$ as the weight function.

Second, $\omega_1(k,l)$ is given as

$$\omega_1(k,l) = \begin{cases} 0 & , \text{ if } g(k,l) \le p(k,l), \\ \dfrac{\left|\mathbf{F}_{:,k}^{\mathrm{H}}(\mathbf{R} \odot \mathbf{T}_l)\right|}{\sqrt{I(k,l)}} & , \text{ otherwise}, \end{cases} \tag{9}$$

where $g\,(k,l) := \left|\mathbf{F}_{:,k}^{\mathrm{H}}(\mathbf{R} \odot \mathbf{T}_l)\right|^2 - I(k,l) + I(k,l)\log(\frac{I(k,l)}{\left|\mathbf{F}_{:,k}^{\mathrm{H}}(\mathbf{R} \odot \mathbf{T}_l)\right|^2})$, which is non-negative. Furthermore, $g(k,l) = 0$ when $\mathbf{R}$ is the ground truth, and $p(k,l)$ is a given parameter which can be adjusted according to different situations. In turn, also the reasoning behind (9) is based on two arguments. First, since actually measured are counts of photons arriving at the APDs, i.e., integers, the error caused by the truncation must be considered. As a result, we require $\left|\mathbf{F}_{:,k}^{\mathrm{H}}(\mathbf{R} \odot \mathbf{T}_l)\right|^2$ to be in the vicinity of $I(k,l)$ such that $g(k,l) \le p(k,l)$ in order to overcome overfitting. At the same time, the value of $p(k,l)$ also depends on $I(k,l)$. Specifically, $p(k,l)$ can take a larger value if $I(k,l)$ has more counts. As a result, the bound of $g(k,l)$ which makes $\omega_1(k,l) = 0$ gets broader. Consequently, more tolerances will be considered when fitting the (k,l)th element of $\mathbf{I}$. For numerically simulated 2D input data sets, $p(k,l)$ can be approximated as 0. Second, when $g\,(k,l) > p(k,l)$, we have $\omega_1(k,l) = \frac{\left|\mathbf{F}_{:,k}^{\mathrm{H}}(\mathbf{R} \odot \mathbf{T}_l)\right|}{\sqrt{I(k,l)}}$. As discussed in Ref. [4], $\omega_1(k,l)$ can be viewed as the confidence score on the reliability or meaningfulness of the corresponding gradient, so here we use it as a factor of the weight function $\omega(k,l)$ to measure the importance of the loss terms.

E. Acceleration and restart

To accelerate the numerical convergence, we use an momentum acceleration algorithm [5] at each iteration, namely,

$$\begin{aligned} \tilde{\mathbf{R}}^{(m)} &= \mathbf{R}^{(m-1)} - \mu \nabla \ell_\omega \left(\mathbf{R}^{(m-1)}\right) , \\ \mathbf{R}^{(m)} &= \tilde{\mathbf{R}}^{(m)} + \tfrac{t-1}{t+2} \left(\tilde{\mathbf{R}}^{(m)} - \tilde{\mathbf{R}}^{(m-1)}\right) , \end{aligned} \tag{10}$$

SUPPLEMENTARY TABLE I. Baseline FLOPS counts at each iteration of the NPRS algorithm. Counts of $O(1)$ and pre-computations have been omitted.

Per computation iteration	FLOPS
$\nabla \ell_\omega \left(\mathbf{R}^{(m-1)}\right)$	$2Ln\text{log}n + 2KLn + 2nL + 12KL$
$\mathbf{R}^{(m)}$ via formulation	$5n$
update auxiliary t	$nL + Ln\text{log}n + 8KL$

where the gradient $\nabla \ell_\omega (\mathbf{R})$ is calculated as below:

$$\nabla \ell_\omega (\mathbf{R}) = \sum_{k=1}^{K} \sum_{l=1}^{L} \omega(k,l) \left(\frac{\mathbf{F}^{\mathrm{H}}_{:,k}(\mathbf{R} \odot \mathbf{T}_l) \left(\left| \mathbf{F}^{\mathrm{H}}_{:,k}(\mathbf{R} \odot \mathbf{T}_l) \right|^2 - I(k,l) \right)}{\left| \mathbf{F}^{\mathrm{H}}_{:,k}(\mathbf{R} \odot \mathbf{T}_l) \right|^2} \right) \left(\mathbf{F}_{:,k} \odot \mathbf{T}^{\mathrm{H}}_l \right). \quad (11)$$

With acceleration, the gradient method may converge as fast as $\mathcal{O}(1/m^2)$ in the optimal case. At the same time, because the problem is also non-convex, we use the restart technique to make the acceleration more robust and prevent the algorithm to stagnate easily. Specifically, if $\ell_\omega(\mathbf{R}^{(m)})$ does not decrease sufficiently, namely, $\ell_\omega(\mathbf{R}^{(m)}) \geq \ell_\omega(\mathbf{R}^{(m-1)})$, it indicates that the result of the current iteration isn't satisfactory. Thus, in this case we choose to reduce the influence caused by the momentum in the next iteration and set $t = 1$.

F. Computational costs of the NPRS algorithm

The computational complexity of the NPRS algorithm is dominated by matrix multiplications in calculating the gradient and the weights, see for instance Eqs. (9) and (11). Other steps only add a negligible amount of computation cost. The details are shown in Table I. The total computational cost of each step is approximated as $5n + 3nL + 2KLn + 20KL + 3Ln\text{log}n$ floating operations per second (FLOPS).

Next, simulations were implemented to illustrate the performance of the NPRS algorithm using the acceleration module compared with vanilla gradient. We chose again as starting point the 2D data set simulating nuclear forward scattering with aligned sample magnetization discussed in the main text. The total iteration number was chosen for both cases to be $50,000$. The common initialization is $\mathbf{1}$. Other parameters were the same with the simulations in Fig. 2. Similarly, the computational cost at each iteration for NPRS with vanilla gradient is $2n + 2nL + 2KLn + 4KL + 2Ln\text{log}n$. The results are shown in Fig. 3. We find that the NPRS algorithm combined with the acceleration modules can improve the

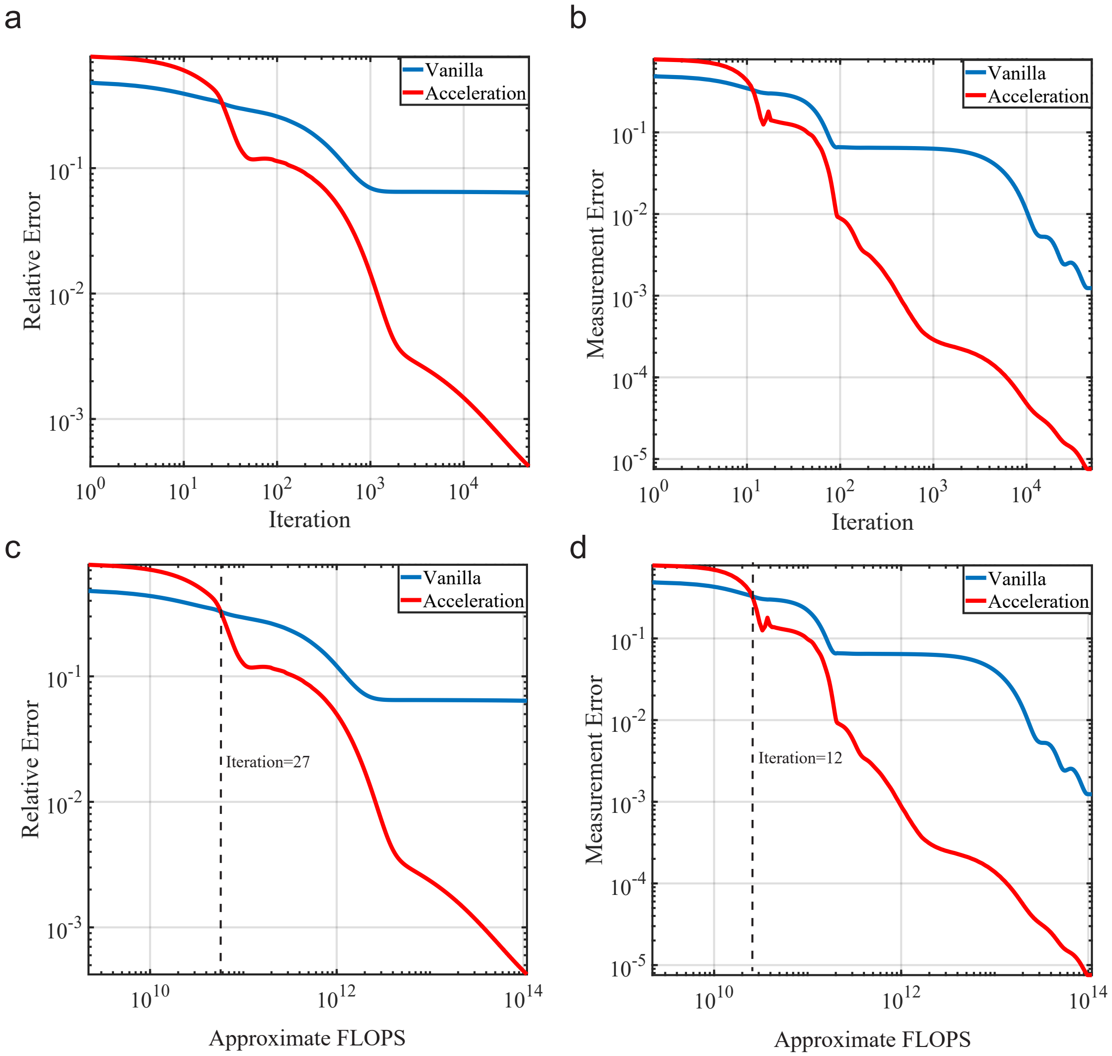

SUPPLEMENTARY FIG. 3. **Vanilla gradient and acceleration modules.** **a** Relative and **b** measurement errors for spectra recovered by the NPRS algorithm using the vanilla gradient and acceleration modules as a function of the algorithm iteration. **c** Relative and **d** measurement errors as a function of computation time (FLOPS). The retrieval was performed on the same simulated 2D data set used for Fig. 2.

convergence speed. At the same time, although the acceleration module introduces extra computational cost, we find that it actually demands fewer total computations to achieve a lower error as apparent from Fig. 3**c-d**.

NUMERICAL TESTS FOR THE NPRS ALGORITHM

In this Section, we perform and discuss several numerical tests concerning the capabilities and performance of the NPRS algorithm. First, we test its universality by investigating its

performance on several other resonant nuclear x-ray scattering setups, in all cases considering as input numerically generated 2D spectra. Then, we proceed to investigate the role of the analyzer thickness for the NPRS algorithm and show that its accuracy is fairly stable across a large range. Finally, we implement and compare several state-of-the-art ptychography methods for phase and energy spectrum retrieval. Based on the four experimental setups discussed throughout this work, we show that our NPRS is faster and more accurate than several ptychography algorithms.

G. Universality of the NPRS algorithm

To test the universality of our NPRS algorithm, we apply it to 2D input spectra simulated considering several different setups. In addition to the nuclear forward scattering setup with aligned magnetization discussed in the main text, three scenarios of resonant nuclear x-ray scattering which have been demonstrated experimentally so far are considered:

(i) A nuclear forward scattering setup where a linearly polarized x-ray pulse irradiates the target containing α-Fe in free space. When the target is randomly magnetized, six transitions of different energies occur in the spectrum. In comparison, the setup investigated in the main text addresses the particular case in which a weak external magnetic field aligns the magnetization orientation of the target such that only two transitions are driven. Results for this case are presented in Fig. 4.

(ii) The setup recently demonstrated in Ref. [6] combining a nuclear forward scattering geometry with aligned sample magnetization (only $\Delta m = 0$ transitions are driven) with fast mechanical motion of the target which reshuffles the spectral components of the x-ray pulse. See results in Fig. 5 and discussion below.

(iii) A thin-film cavity setup in grazing incidence. The incoming x-ray pulse couples to the cavity evanescently. The generated cavity field excites the nuclei in a ^{57}Fe layer embedded in the cavity. For the simulations we have used the structure Ta (2 nm)/B (17 nm)/^{57}Fe(3 nm)/B (7 nm)/Ta (9 nm) (similar to the ones in Refs. [7, 8]) and the x-ray pulse incidence angle 3.5 mrad. As in the case of (ii), the sample has aligned sample magnetization, such that only the two $\Delta m = 0$ transitions are considered. The input and retrieved data sets are presented in Fig. 6.

The simulated response function $R(\Delta)$ is obtained for (i) and (ii) by using analytical expressions from the literature [9]. For the thin-film cavity setup (iii), we use a numerical implementation of the Parratt formalism that we have benchmarked with the software packages CONUSS [10] and Pynuss. For each case, the 2D spectra are numerically generated using Eq. (2) and the analyzer transmission function fitted from the experiment (analyzer thickness of 1 μm) and given in the main text. For the algorithm initialization, we use $\alpha\mathbf{I}$, and a total number of iterations of 20,000. We follow the analysis in the main text and retrieve the intensity and phase spectra as a function of energy, reconstruct the 2D data set, and calculate the relative and measurement errors. For comparison, calculated TIS and PDTD spectra are also presented. The quality of the recovered TIS spectra depends on the integration window and the thickness of the analyzer. In order to obtain the best possible results, we optimize the integration range for each example separately, however using the same analyzer parameters (for which we consider here the parameters from the experiment described in the main text). We find that for all considered cases, the NPRS algorithm delivers accurate spectra and displays lower measurement errors than PDTD.

H. Robustness tests under noise corruption

In this Subsection, we will assess the robustness of the NPRS algorithm for noise-corrupted spectra. Since the photon counts in our calculations are assumed to follow a Poisson distribution, we accordingly consider Poisson noise. In the simulations, we numerically generate the 2D spectra of the 4 cases discussed above using Eq. (2) and the analyzer transmission function fitted from the experiment (analyzer thickness of 1 μm), as detailed in the main text. For each Δ_{D}, the photon counts $I(k,l)$ are corrupted by Poisson noise with 3 values of signal to noise ratio (SNR), specifically 20 dB, 45 dB and 60 dB. Subsequently, the NPRS algorithm is applied to recover the signal of interest from these corrupted simulations. Denoting the signal by $\mathbf{I} \in \mathbb{R}^n$, and the noise by $\boldsymbol{\omega} \in \mathbb{R}^n$, the SNR is defined as $20 \log \frac{\|\mathbf{I}\|}{\|\boldsymbol{\omega}\|}$.

We record for each iteration the relative and measurement errors. As discussed in the main text, a lower measurement error does not necessarily indicate better recovery performance due to the danger for overfitting noise. This is why we stop the algorithm iterations once the relative error reaches a minimum. The results are shown in Figs. 7-10, which demonstrate that our approach exhibits robustness to noise. At SNR levels above 60 dB, both the

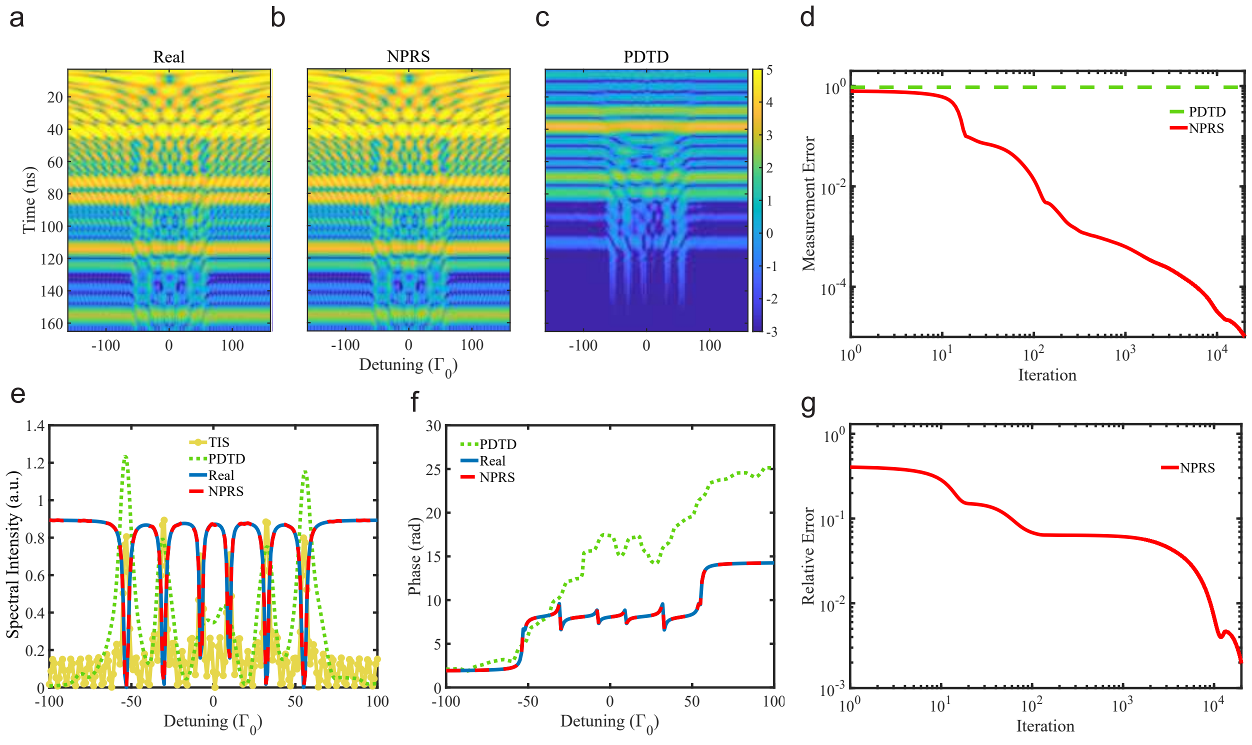

SUPPLEMENTARY FIG. 4. **Numerical results for an α-Fe sample of 2.3 μm with random magnetization.** **a** Input 2D time- and energy-dependent intensity data set, simulated numerically. **b**, **c** Reconstructed time- and energy-dependent spectra using the NPRS algorithm and PDTD, respectively. **d** Measurement error as a function of iteration steps. **e** Recovered intensity using NPRS, PDTD and TIS. **f** Recovered phase using NPRS and PDTD. **g** Relative error for NPRS as a function of algorithm iterations.

intensity spectra and phase are accurately recovered. As SNR decreases, small oscillations appear in the intensity spectra, yet the overall profile remains intact. Remarkably, the step-wise profile of the phase remains highly stable even at an SNR of 20 dB. The very sharp phase jumps at approx. $\pm 31.5\Gamma_0$ are still recovered for a SNR of 60 dB, but start to blurr out for higher noise values.

In summary, both intensity and phase spectra exhibit robustness to Poisson noise, with the phase demonstrating high stability, up to the two sharp phase jumps which are lost at noise above 60 dB SNR. This underlines the advantage of our method in effectively recovering phase information.

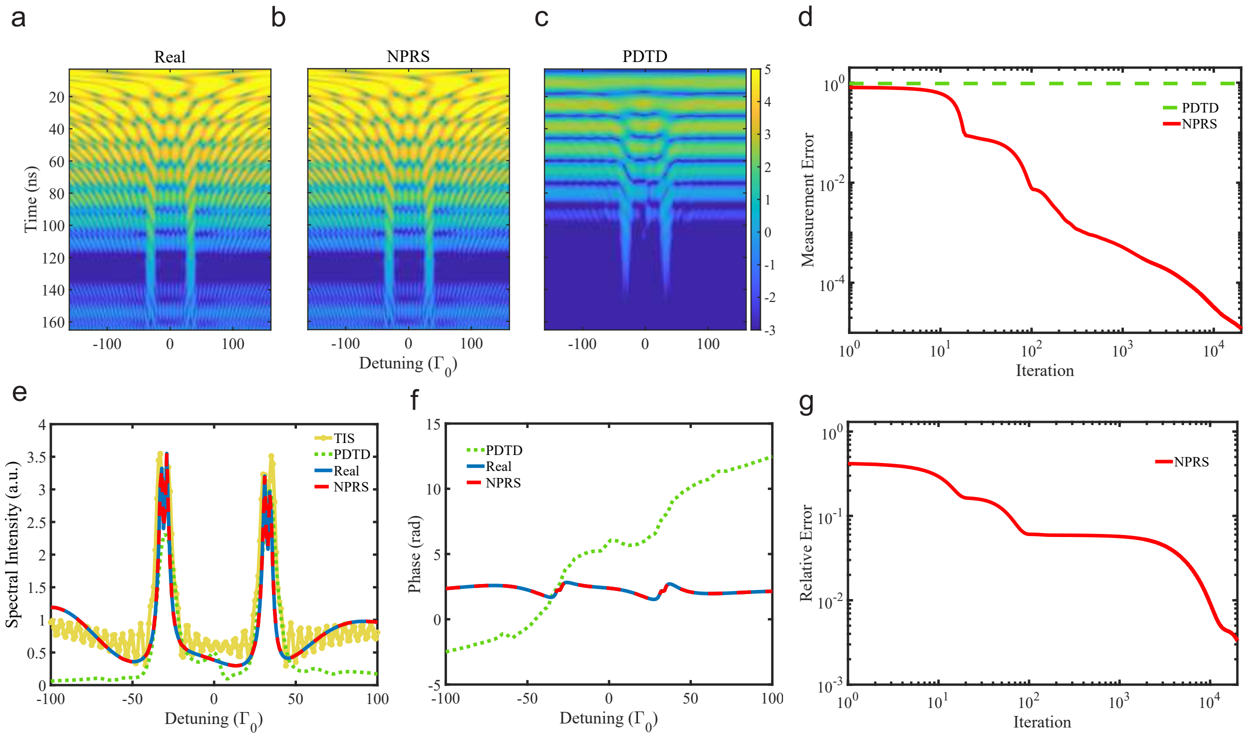

SUPPLEMENTARY FIG. 5. **Numerical results for a fast mechanical motion with the displacement 0.43 Å is applied on a α-Fe sample of 2.0 μm with aligned magnetization.** **a** Input 2D time- and energy-dependent intensity data set, simulated numerically. **b**, **c** Reconstructed time- and energy-dependent spectra using the NPRS algorithm and PDTD, respectively. **d** Measurement error as a function of the iteration steps. **e** Recovered intensity using NPRS, PDTD and TIS. **f** Recovered phase using NPRS and PDTD. **g** Relative error for NPRS as a function of algorithm iterations.

I. Role of analyzer thickness

So far, we have considered for all calculations an analyzer effective thickness of 1 μm, which is the value of the actual analyzer used in the reported experiment. In this subsection, we address the role of the analyzer thickness for the performance of the NPRS algorithm. To this end, we consider analyzers with different effective thickness for the numerical simulations. We consider a single-resonance $K_2Mg^{57}Fe(CN)_6$ analyzer with the effective thickness d. We recall that for all samples and analyzers we consider enriched ^{57}Fe iron, such that the effective thickness corresponds to the actual sample thickness. For the normal incidence setups, a larger thickness of sample or analyzer will correspond to a broader linewidth.

The response function is retrieved for the four cases addressed in this work in the main manuscript and in Section G: nuclear forward scattering with aligned and random magne-

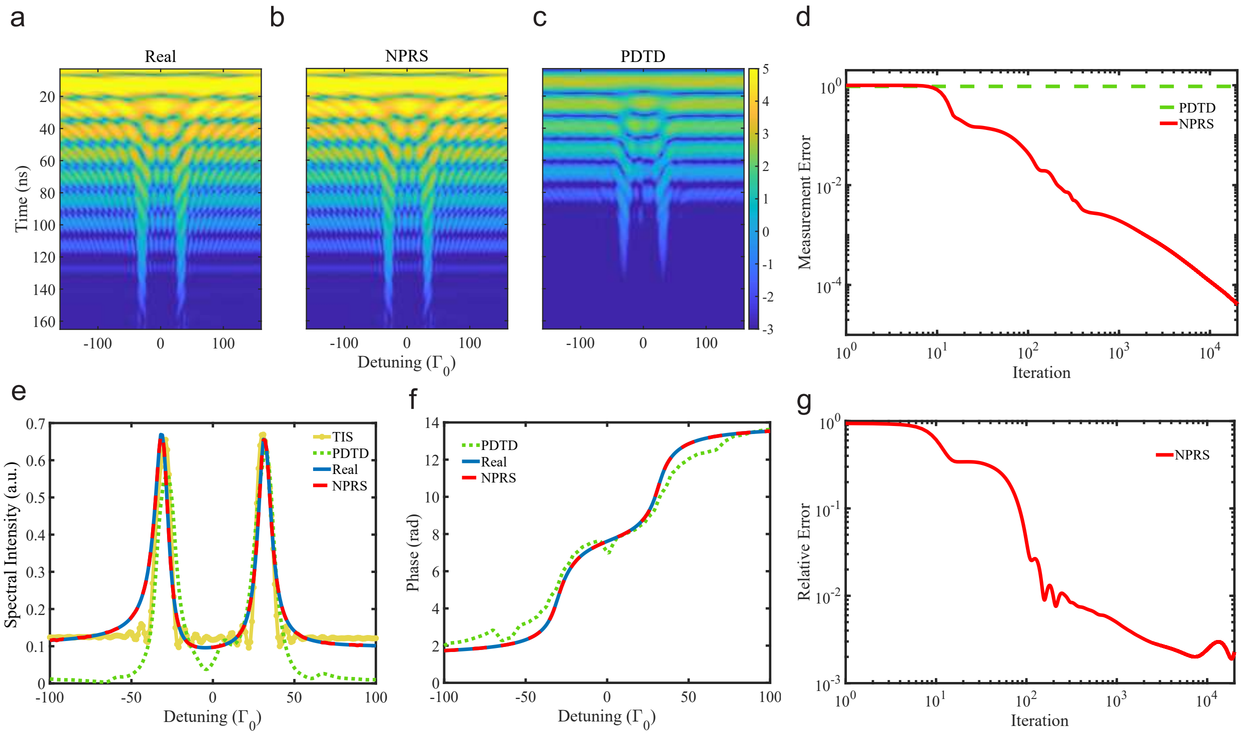

SUPPLEMENTARY FIG. 6. **Numerical results for a thin-film cavity with the structure Ta (2 nm)/B (17 nm)/^{57}Fe(3 nm)/B (7 nm)/Ta (9 nm) at the incidence angle** 3.5 **mrad. a** Input 2D time- and energy-dependent intensity data set, simulated numerically. **b**, **c** Reconstructed time- and energy-dependent spectra using the NPRS algorithm and PDTD, respectively. **d** Measurement error as a function of the iteration steps. **e** Recovered intensity using NPRS, PDTD and TIS. **f** Recovered phase using NPRS and PDTD. **g** Relative error for NPRS as a function of algorithm iterations.

tization, with mechanical motion, and thin-film cavities in grazing x-ray incidence. For each setup, the corresponding 2D intensity spectrum **I** is generated by Eq. (2) using the same sample parameters as in Section G but with analyzer response function **T** corresponding to different analyzer thickness values ranging from 0.5μm to 5μm. The analyzer transmission function is calculated in this case using analytical expressions.

The analyzer thickness produces visible changes in the simulated input 2D sets, as can be seen in an example in Fig. 11 which presents in the first two columns results for a 1 μm- and a 3 μm-thick analyzers. For the results presented in Fig. 11, the NPRS algorithm is applied using once more $\alpha\mathbf{I}$ as initialization and 20,000 iterations. The reconstructed 2D data set after NPRS has a much better quality than the PDTD reconstruction, presented for comparison.

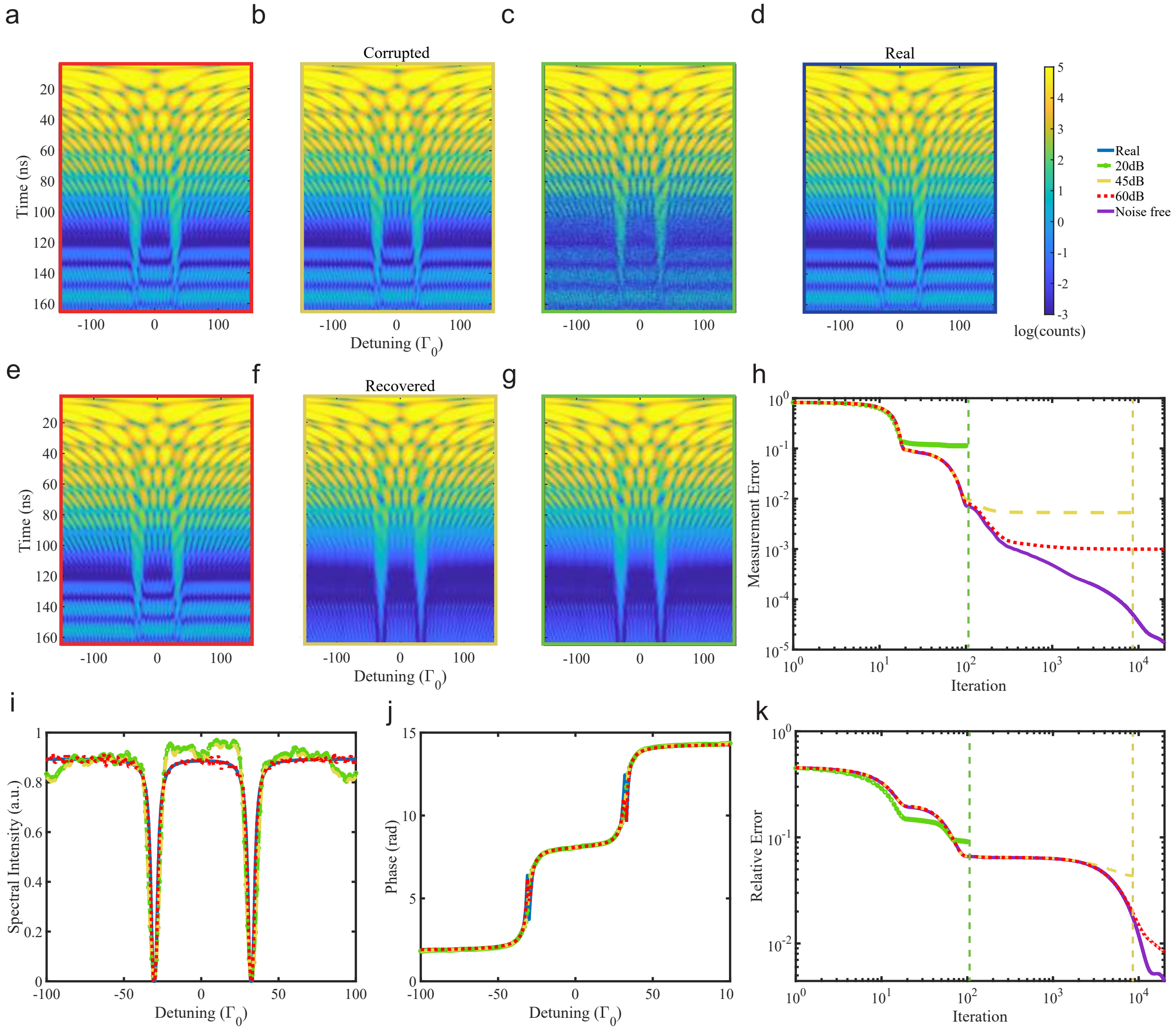

SUPPLEMENTARY FIG. 7. **Numerical results for the α-Fe enriched sample of 2.3 μm with an aligned magnetization and an analyzer of 1 μm effective thickness.** **a-c** are the corrupted simulations with SNR 20 dB, 45 dB and 60 dB respectively. **d** is the original simulation without noise. **e-g**, and **i-j** show the recovered 2D spectrum, spectral intensities and phases. **h** and **k** record the relative errors and measurement errors of different circumstances.

For a more quantitative analysis, we proceed to calculate the measurement errors and the relative errors for the retrieved spectra of all four considered setups as a function of iteration and of analyzer thickness, where we consider the range 0.5μm to 5μm. The results are displayed in Fig. 12. We find that all measurement errors are below 10^{-4} and relative errors below 10^{-2}, respectively. The measurement errors are displaying very similar values for all considered analyzer thicknesses. While for the relative error for some setups a thicker analyzer appears to deliver a smaller value, the overall differences throughout the thickness

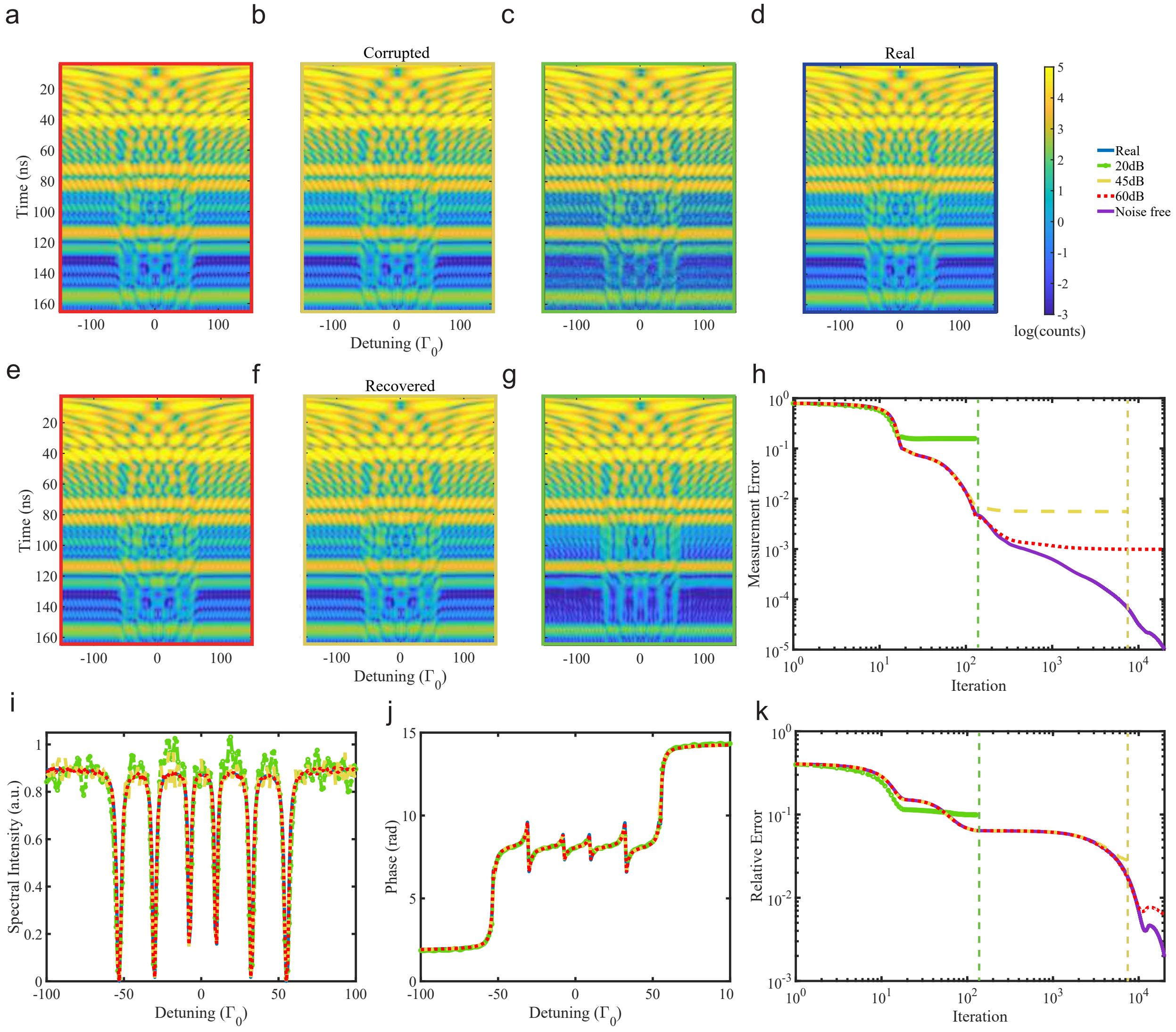

SUPPLEMENTARY FIG. 8. **Numerical results for an α-Fe enriched sample of 2.3 μm with random magnetization.** **a-c** are the noise-corrupted simulations with SNR 20 dB, 45 dB and 60 dB respectively. **d** is the original simulation without noise. **e-g**, and **i-j** show the recovered 2D spectrum, spectral intensities and phases. **h** and **k** record the relative errors and measurement errors of different circumstances.

range remain small. We conclude that the NPRS algorithm is very robust with respect to the analyzer thickness, which is a clear advantage as opposed to other methods such as TIS and PDTD.

Finally, we may compare the energy resolution of the NPRS algorithm with the one of a Synchrotron Mössbauer Source (SMS), which enables conventional (energy-domain) Mössbauer spectroscopy at SR facilities. The key element for an SMS is the iron borate crystal $^{57}FeBO_3$ whose properties determine the energy resolution of the SMS. For a com-

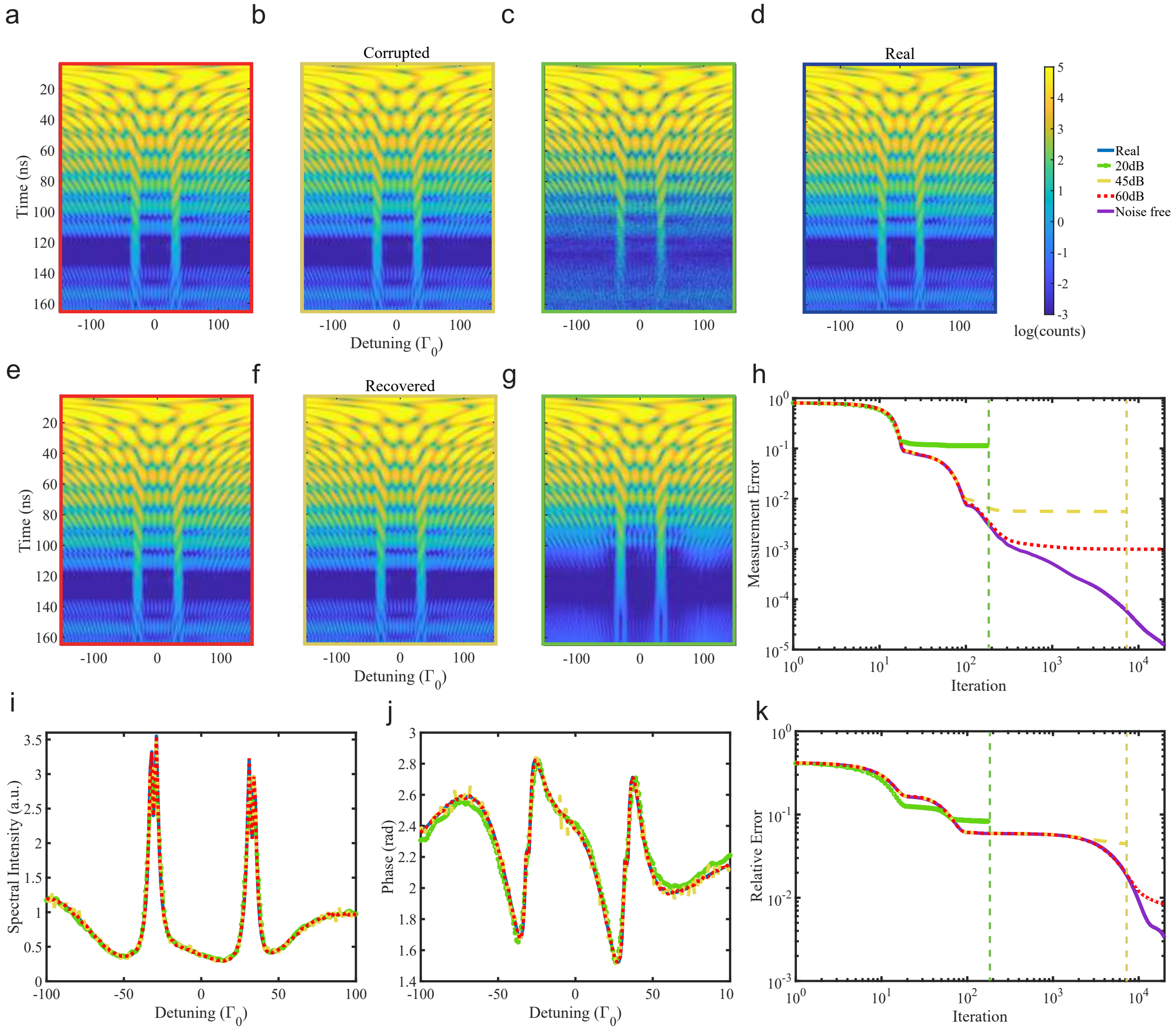

SUPPLEMENTARY FIG. 9. **Numerical results for the case when a fast mechanical motion with the displacement** $0.43\,\text{Å}$ **is applied on an** α**-Fe enriched sample of** $2.0\,\mu$**m with aligned magnetization.** **a-c** are the noise-corrupted simulations with SNR 20 dB, 45 dB and 60 dB respectively. **d** is the original simulation without noise. **e-g**, and **i-j** show the recovered 2D spectrum, spectral intensities and phases. **h** and **k** record the relative errors and measurement errors of different circumstances.

parison, starting from a known response function $\mathbf{R}(\Delta)$ of a sample, we simulate numerically an SMS spectrum by convoluting $\mathbf{R}(\Delta)$ with a Lorentzian of width Γ, which describes the energy resolution of the SMS. As before, for the NPRS results we simulate the 2D input spectrum with a 1 μm analyzer thickness, and retrieve the response function. The comparison is presented in Fig. 13 for two SMS energy resolution values $\Gamma = 2.2\Gamma_0$, $6.6\Gamma_0$ (which are examples in a typical range of experimental SMS parameters [11]) for all four considered

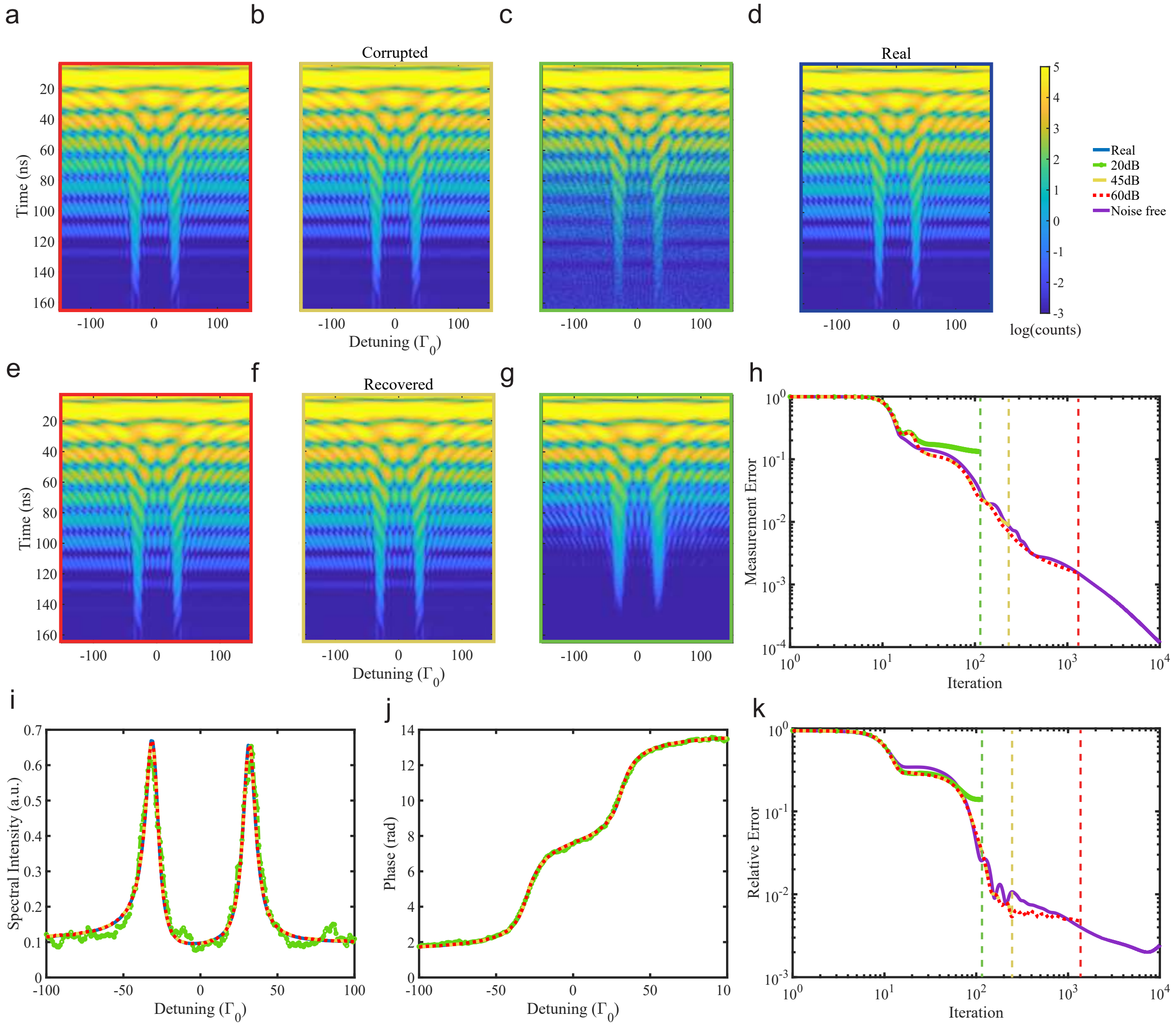

SUPPLEMENTARY FIG. 10. **Numerical results for a thin-film cavity with the structure Ta (2 nm)/B (17 nm)/**^{57}Fe**(3 nm)/B (7 nm)/Ta (9 nm) at the incidence angle** 3.5 **mrad.** **a-c** are the corrupted simulations with SNR 20 dB, 45 dB and 60 dB respectively. **d** is the original, noise-free simulation. **e-g**, and **i-j** show the recovered 2D spectrum, spectral intensities and phases. **h** and **k** record the relative errors and measurement errors of different circumstances.

setups. While the NPRS results present an excellent agreement with the original response function, the SMS results are less accurate, and especially for $\Gamma = 6.6\Gamma_0$ visible deviations appear.

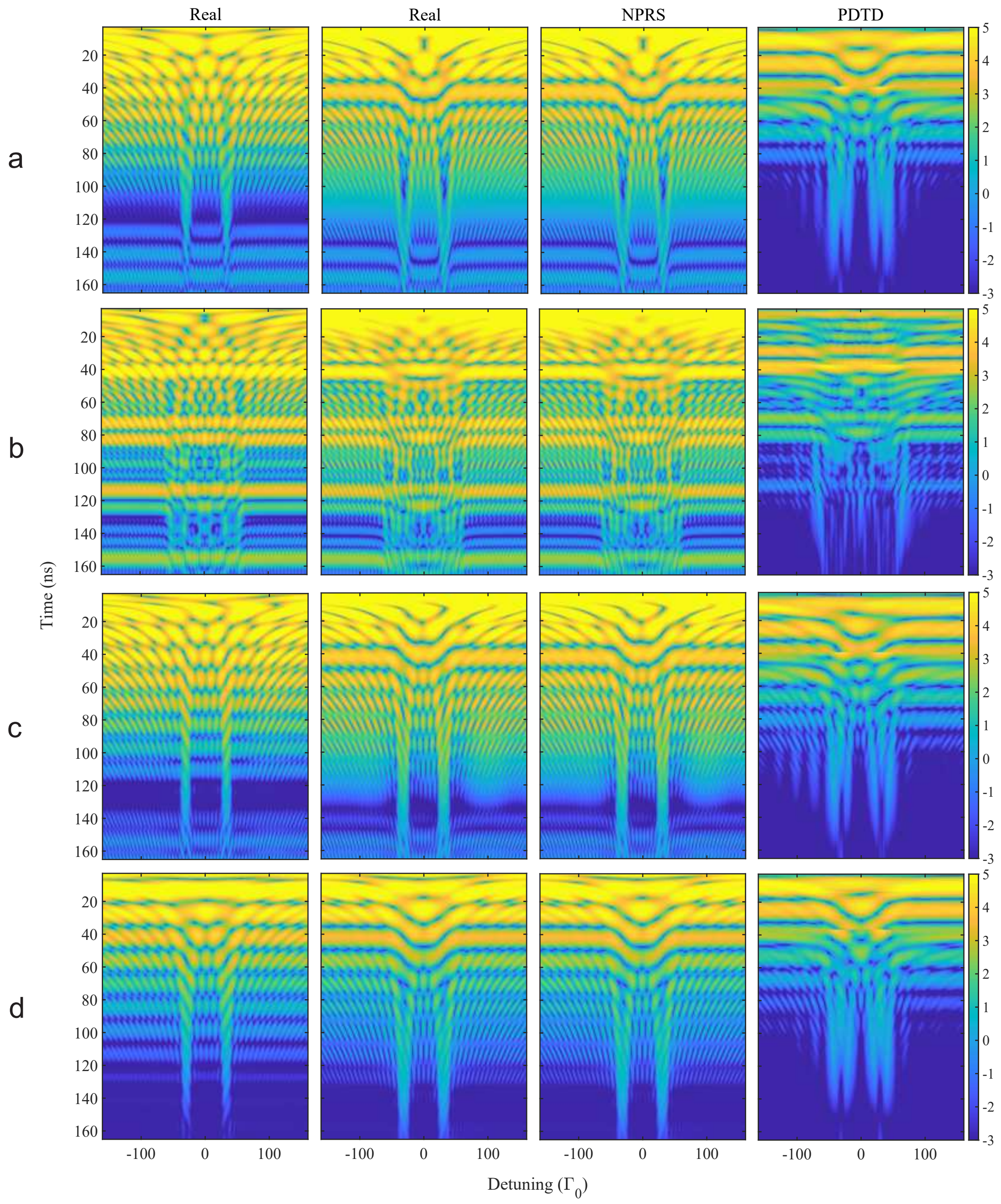

SUPPLEMENTARY FIG. 11. **Simulated 2D data sets.** **a** Nuclear forward scattering for $\Delta m = 0$ transitions **b** random magnetization, **c** nuclear forward scattering for $\Delta m = 0$ and mechanical motion, **d** the thin-film cavity setup examples discussed in Section G. The 2D spectra in the first column are generated by the analyzer with the thickness $d \approx 1\mu$m, whereas for the second column we have used an analyzer with the thickness $d \approx 3\mu$m. The recovered 2D spectra for the case of $d \approx 3\mu$m using the NPRS algorithm and PDTD are presented in the third and fourth columns, respectively.

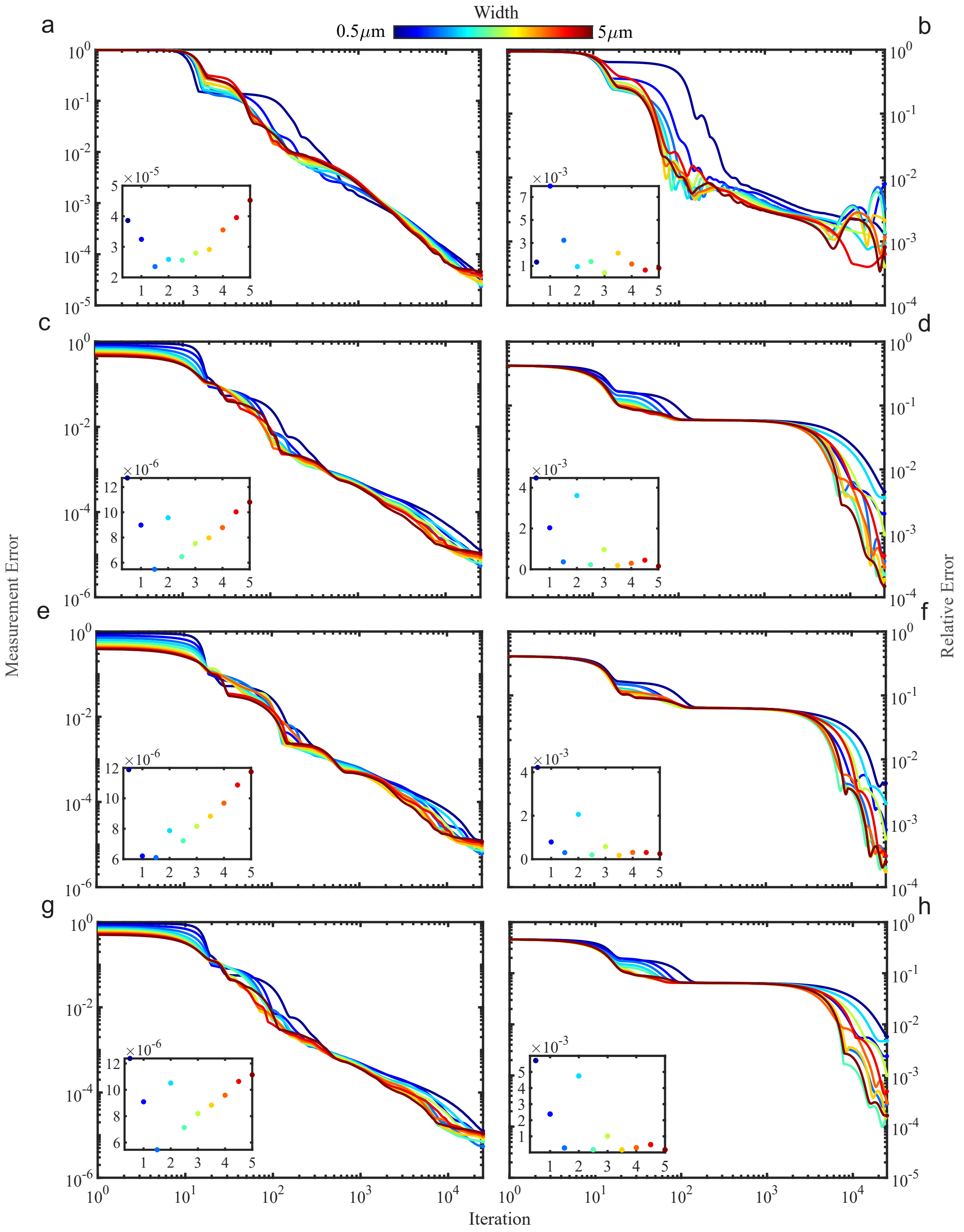

SUPPLEMENTARY FIG. 12. **Measurement errors and relative errors for spectra recovered with the NPRS algorithm for analyzer thickness values in the ranges (0.5 - 5) μm.** The considered setups are once more **a, b** nuclear forward scattering for $\Delta m = 0$ transitions **c, d** nuclear forward scattering with random magnetization, **e, f** nuclear forward scattering for $\Delta m = 0$ and mechanical motion and **g, h** the thin-film cavity setup. All examples consider the same parameters introduced already in Section G.

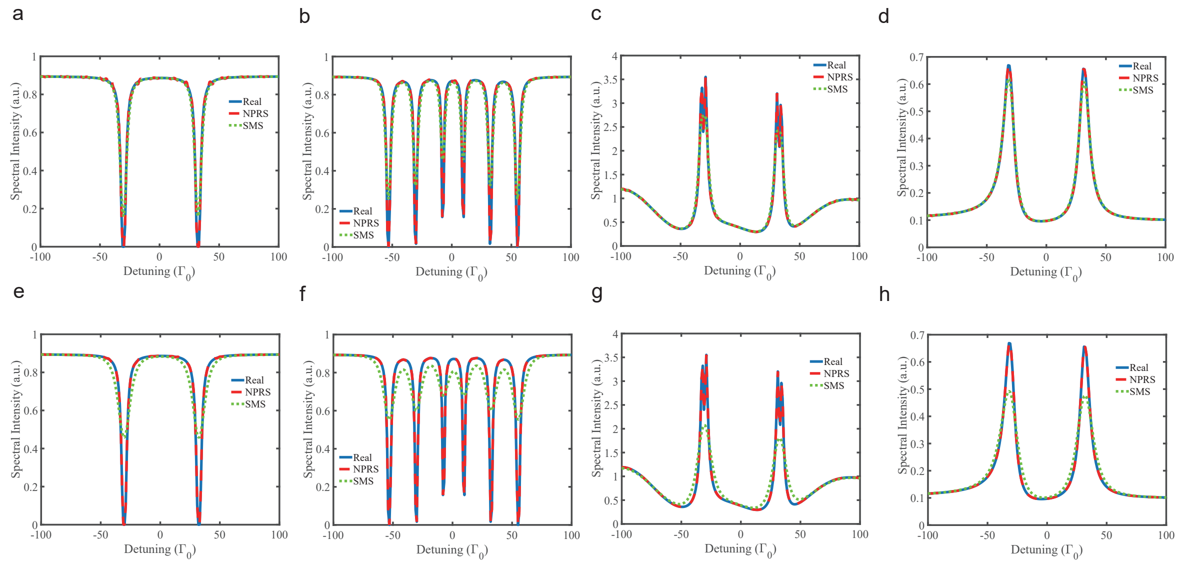

SUPPLEMENTARY FIG. 13. **Simulated SMS and retrieved NPRS intensity spectra as a function of detuning.** We compare to the original response function $\mathbf{R}(\Delta)$ labelled as "Real". The considered setups are once more **a, e** nuclear forward scattering for $\Delta m = 0$ transitions **b, f** nuclear forward scattering with random magnetization, **c, g** nuclear forward scattering for $\Delta m = 0$ and mechanical motion and **d, h** the thin-film cavity setup. All examples consider the same parameters introduced already in Section G. The upper (lower) row SMS spectra were obtained with an energy resolution of $2.2\Gamma_0$ ($6.6\Gamma_0$).

J. Comparison with different ptychography methods

The problem described in Eq. (6) can be considered a generalization of ptychography [12]. In the following, we discuss two essential differences between the two approaches. First, the formulations of the two methods are different. In the case of the NPRS algorithm, for each Δ_D the entire energy spectrum $\mathbf{R}$ is convoluted with the analyzer $\mathbf{T}$ in Eq. (6). In contrast, for traditional ptychography models, $\mathbf{R}$ is divided into several overlapping regions, then each region is considered separately. Second, Eq. (6) is usually more ill-posed than the traditional ptychography model, as n is typically larger than K in practice. This results in under-sampling for Eq. (6), making it more difficult to be handled.

In this subsection, we compare the results of the NPRS algorithm with retrieved spectra using several classical ptychography methods, including PIE (Ptychography Iterative Engine) [13], DR (Douglas Rachford) method [14], and ADMM (Alternating Direction Method of Multiplier) [15]. These methods are tested to recover the response function $\mathbf{R}$ for the four different nuclear resonant x-ray scattering discussed above, with the same initialization of

$\alpha\mathbf{1}$. For all the investigated ptychography methods we use the parameters suggested in their respective references. The total number of iterations for all methods is 20,000. At each iteration, the relative error and measurement error of the estimates are calculated. Additionally, the total computational time consumed by each method is recorded. The results are shown in Figs. 14-17. The comparison is performed for all four setups discussed in Section G.

For all cases, the results recovered by the DR and ADMM method are comparable, while the PIE method diverges before reaching the maximal number of iterations. The similar performance of DR and ADMM has been already discussed in the literature, as the methods are equivalent under some circumstances [16]. For all setups, the NPRS algorithm demonstrates a superior performance compared to all considered ptychography algorithms. In Figs. 15 and 16, the transmission $\hat{\mathbf{R}}$ recovered by the NPRS algorithm has the fewest oscillations when fitting $\mathbf{R}$. Additionally, the estimation $\hat{\mathbf{R}}$ recovered by the NPRS algorithm has the lowest measurement and relative errors, which confirms that the NPRS performs best among all methods. Furthermore, NPRS is more efficient and delivers $\hat{\mathbf{R}}$ with the lowest cost in terms of computational time. We conclude that for the nuclear spectroscopy setups discussed in this paper, the NPRS algorithm is preferable to the ptychography algorithms since it is more likely to converge to the ground truth in the least amount of time.

EXPERIMENTAL RESULTS FOR GRAZING INCIDENCE

A second data set was taken during the same beamtime at BL09XU of SPring-8 in Japan for a thin-film cavity setup and grazing incidence of the x-ray pulse. Generally speaking, thin-film x-ray cavities are claddings of several nm-thick layers, starting with a high atomic number Z material, such as Pd or Pt, followed by a low-Z layer and an embedded ^{57}Fe layer. X-ray radiation resonant to the transition from the nuclear ground state to the first excited state couples evanescently to the cavity. Due to the multi-beam interference between the incident and the reflected waves, a standing wave forms at given incidence angles and drives the 14.413 keV transition on the ^{57}Fe nuclear ensemble. Depending on the film thickness, a certain number of cavity guided modes can be excited at different incidence angles where the reflectivity reaches a deep minimum. As the standing wave wavelength is much larger than the nuclear layer thickness, all nuclei experience the same cavity field enabling strong collective effects, such as a shift of the nuclear resonance line known as the collective Lamb

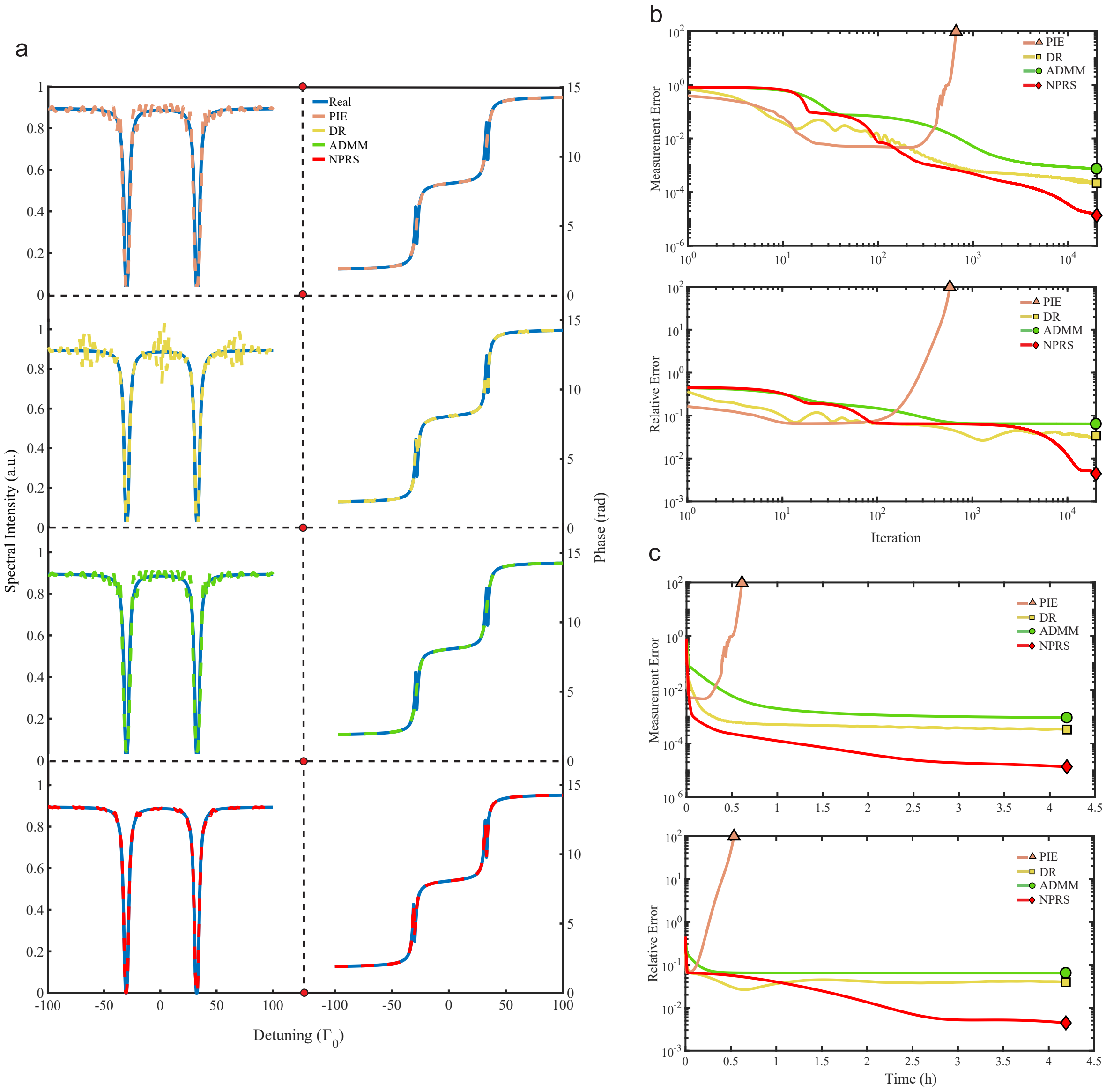

SUPPLEMENTARY FIG. 14. **Comparison between the NPRS and ptychography algorithms for the nuclear forward scattering setup with $\Delta m = 0$ case discussed in Section G.** **a** Spectral intensity and phase recovered by different methods. **b** Relative and measurement errors of the results recovered by different methods at each iteration. **c** Relative and measurement errors as a function of computation time for each method.

shift, and an enhanced superradiant decay rate [17]. The scattered photons are measured as reflectivity at the APD detectors.

The thin-film cavity used in the experiment was deposited on surface polished Si substrates (dimensions $20 \times 20\,\text{mm}^2$) by DC magnetron sputtering technique. A base pressure of 5×10^{-5} Pa was achieved before the cavity layer deposition and pure Argon gas of 0.3

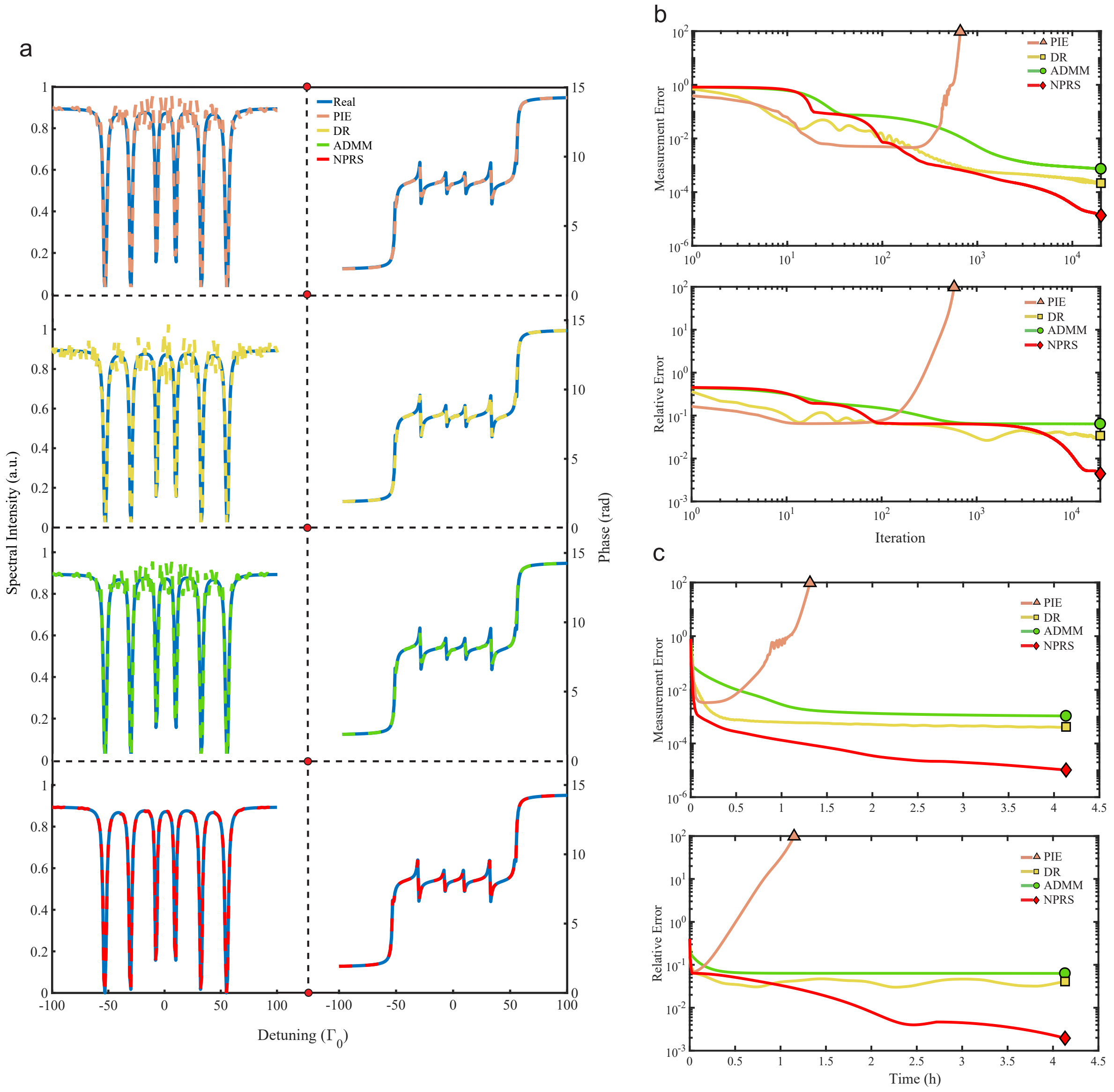

SUPPLEMENTARY FIG. 15. **Comparison between the NPRS and ptychography algorithms for the nuclear forward scattering setup with random sample magnetization discussed in Section G.** **a** Spectral intensity and phase recovered by different methods. **b** Relative and measurement errors of the results recovered by different methods at each iteration. **c** Relative and measurement errors as a function of computation time for each method.

Pa was used as the sputtering working gas for all the deposited layers. For the ^{57}Fe layer, an enriched ^{57}Fe ($\geq$ 95%) ring slightly bigger than the erosion race-track was placed on the natural Iron target. All other layers were deposited by pure element targets. The cavity reflectivity was measured as a function of the incidence angle. Out of this rocking curve, we could fit the cavity parameters obtaining the layer structure Ta_2O_5 (1.5 nm)/Ta (1.8)/B

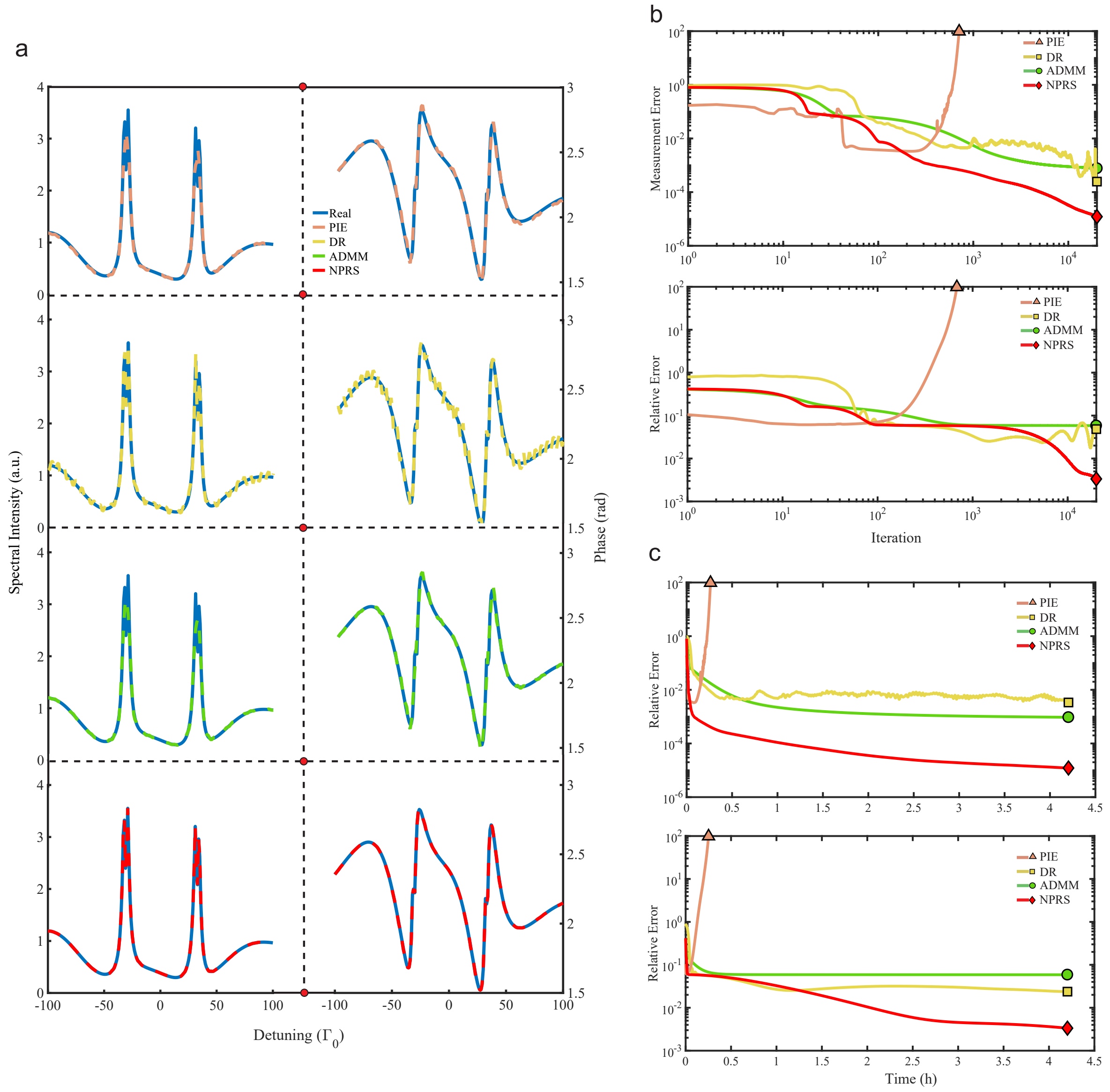

SUPPLEMENTARY FIG. 16. **Comparison between the NPRS and ptychography algorithms for the nuclear forward scattering setup with $\Delta m = 0$ and mechanical motion discussed in Section G.** **a** Spectral intensity and phase recovered by different methods. **b** Relative and measurement errors of the results recovered by different methods at each iteration. **c** Relative and measurement errors as a function of computation time for each method.

(17.2 nm)/^{57}Fe(3.1 nm)/B (6.9 nm)/Ta (8.8 nm)/Si.

For the time- and energy-resolved measurements, we chose the incidence angle $\varphi \approx$ 3.332 mrad, which was very close to the second minimum of the measured rocking curve. In order to align the magnetization of the Fe layers, a weak external magnetic field of approx. 200 mT was used. Also in this setup, only the two $\Delta m = 0$ transitions were driven. The

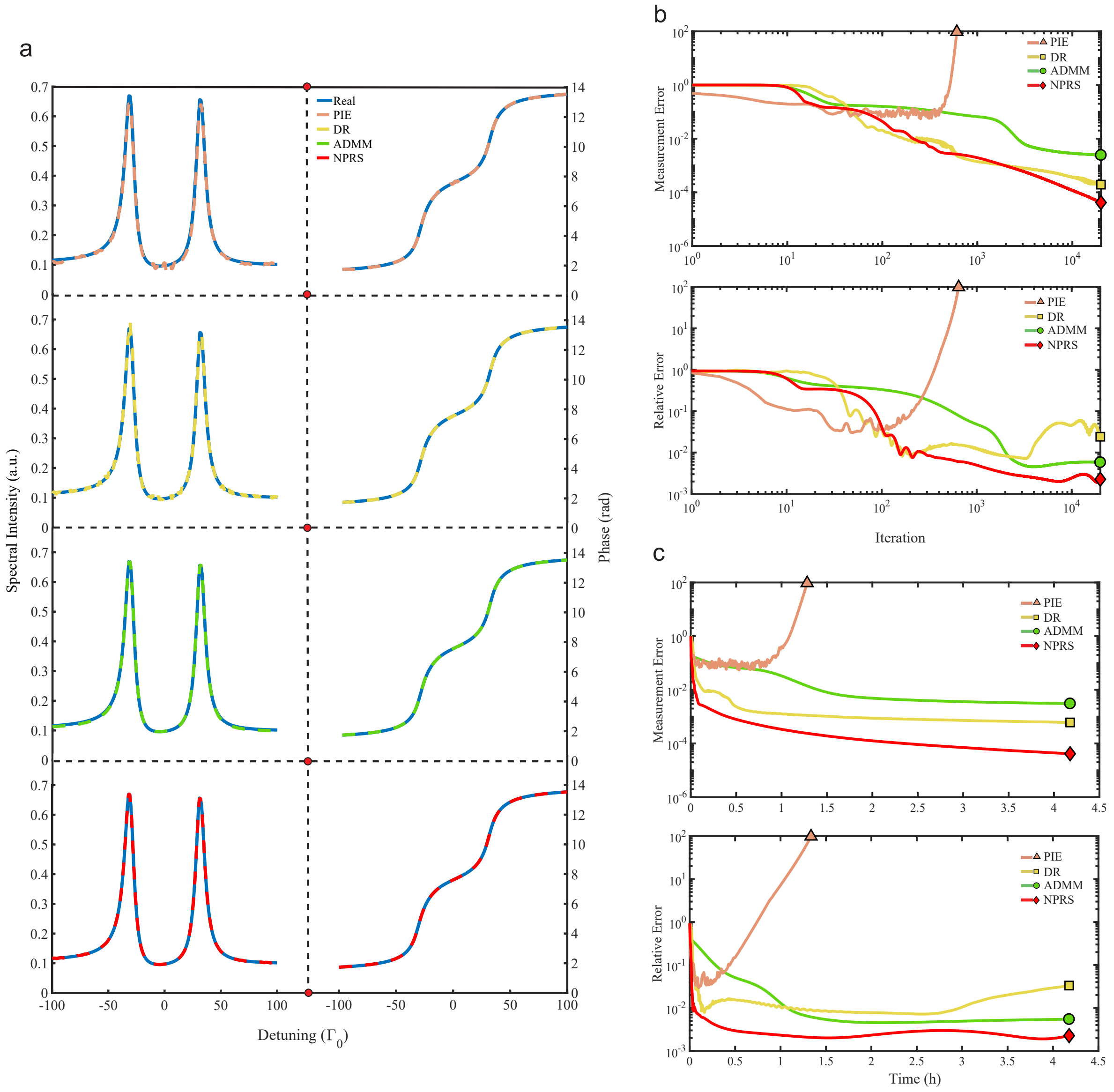

SUPPLEMENTARY FIG. 17. **Comparison between the NPRS and ptychography algorithms for the thin-film cavity setup discussed in Section G. a Spectral intensity and phase recovered by different methods. b** Relative and measurement errors of the results recovered by different methods at each iteration. **c** Relative and measurement errors as a function of computation time for each method.

measured 2D data set is presented in Fig. 18**a**. Unfortunately, due to the poor count statistics, the 2D data set is less satisfactory as in the case of the nuclear forward scattering setup discussed in the main manuscript. A time spectrum was measured independently and is shown in Fig. 18**f**. Also here we notice the presence of noise, most probably related to beam divergence and angle instabilities. Added on the inherent noise introduced by the Doppler

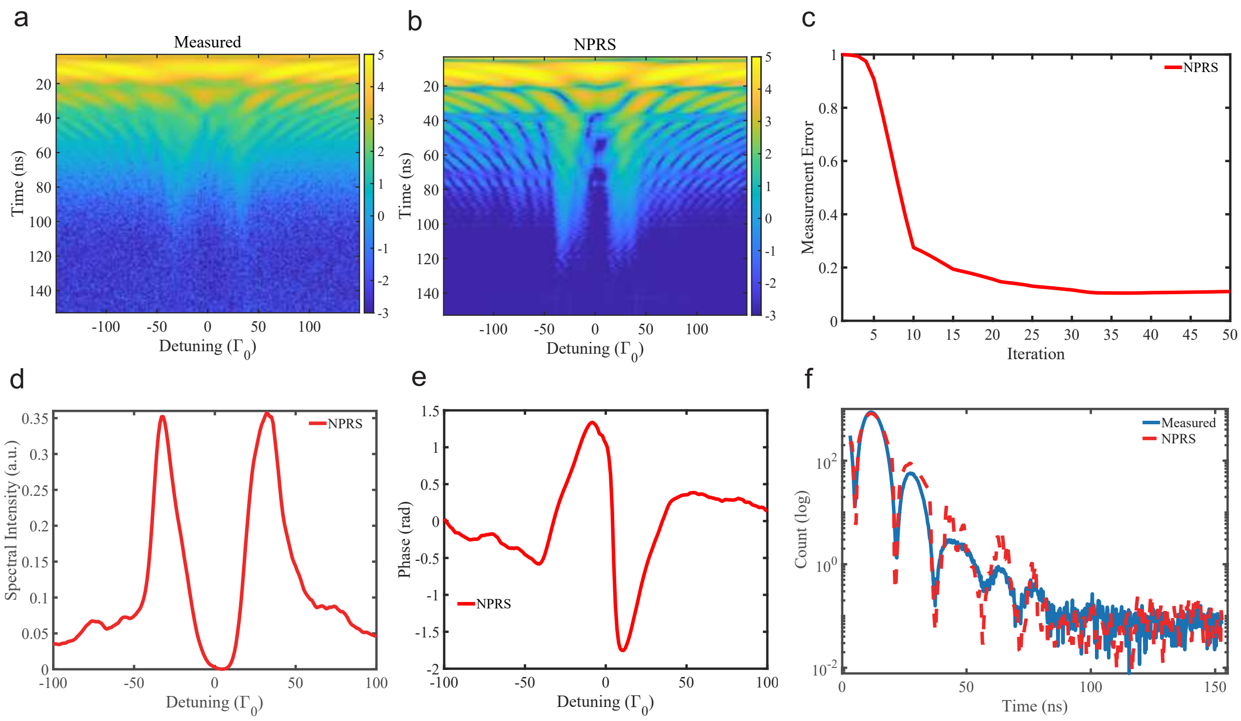

SUPPLEMENTARY FIG. 18. **Experimental and reconstructed results for the thin-film cavity setup. a** Measured 2D intensity spectrum. **b** Reconstructed 2D spectrum. **c** Measurement error for the NPRS algorithm. **d** and **e** demonstrate the recovered spectra with intensity and phase as a function of detuning. **f** Independently measured time spectrum of the sample only, and NPRS spectrum generated by a numerical Fourier transform from the recovered amplitude and phase.

drive in the 2D data spectrum, the overall quality of the taken data sets is lower than for the nuclear foward scattering setup.

Nevertheless, the NPRS algorithm could recover the response function, whos spectral intensity (reflectivity) and phase are presented in Figs. 18**d** and **e**. The expected two-peak structure due to the aligned magnetization setup is visible in the reflectivity spectrum. The reconstructed 2D spectrum is shown in Fig. 18**b**, and the corresponding measurement error in Fig. 18**c**. The latter drops after a small number of iterations to approx. 0.1, showing slightly larger values than the nuclear forward scattering setup.

FURTHER RESEARCH ON ANALYZER-FREE MODEL

The NPRS algorithm assumes the analyzer transmission $\mathbf{T}(\Delta)$ is known in advance. But in some cases, this prior information is not available, leading to a more general but also more

ill-posed problem which remains free of a theoretical model for the analyzer. In the following, we will give more details on the blind algorithms B-NPRS and CB-NPRS and show their performance on numerically simulated and experimental data. A schematic summary on the input and output of the three algorithms NPRS, B-NPRS and CB-NPRS is presented in Fig. 19.

K. Blind Nuclear Phase Retrieval Spectroscopy

1. Formulation of the Mathematical Model

First, we construct the mathematical formulation of the B-NPRS algorithm. Recall that $\mathbf{R}$ and $\mathbf{T}$ are discretized by equally spaced nodes $\Delta_j, j = 0, 1, 2, \cdots, n-1$ with step size Δ_{step}, and the discrete model (2) is formulated as

$$I(t_k, \Delta_D^l) = \left|\mathbf{F}_{:,k}^{\mathrm{H}}(\mathbf{R} \odot \mathbf{T}_l)\right|^2 + \varepsilon, t_k \in \mathbf{E}, \Delta_D^l \in \mathbf{\Omega}.$$

Assume $\Delta_D^1, \Delta_D^2, \cdots, \Delta_D^L$, which are the multiples of Δ_{step}, are ordered decreasingly. Define $\Delta_D^{1^+} = \max(\Delta_D^1, 0)$, $\Delta_D^{L^-} = \min(\Delta_D^L, 0)$, and $h := \frac{\Delta_D^{1^+} - \Delta_D^{L^-}}{\Delta_{\text{step}}}$, then define a vector $\mathbf{T} \in \mathbb{C}^{n+h}$, which has the formulation below

$$\mathbf{T} = \left(T(\Delta_0 - \Delta_D^{1^+}), T(\Delta_0 - \Delta_D^{1^+} + \Delta_{\text{step}}), T(\Delta_0 - \Delta_D^{1^+} + 2\Delta_{\text{step}}), \cdots, T(\Delta_{n-1} - \Delta_D^{L^-})\right).$$

At the same time, we also construct a set of matrices $\{\mathbf{C}_l \in \mathbb{C}^{n\times(n+h)}, l = 1, 2, \cdots, L\}$ as below (the row and column indices are given in blue for orientation),

$$\mathbf{C}_l = \begin{array}{c} \\ 1 \\ 2 \\ \vdots \\ n \end{array} \begin{array}{c} \begin{matrix} 1 & 2 & \cdots & f_l+1 & f_l+2 & \cdots & f_l+n & \cdots & h+n \end{matrix} \\ \begin{pmatrix} 0 & 0 & \cdots & 1 & 0 & \cdots & 0 & \cdots & 0 \\ 0 & 0 & \cdots & 0 & 1 & \cdots & 0 & \cdots & 0 \\ \vdots & \vdots & \cdots & \vdots & \vdots & \ddots & \vdots & \cdots & \vdots \\ 0 & 0 & \cdots & 0 & 0 & \cdots & 1 & \cdots & 0 \end{pmatrix} \end{array},$$

where $f_l = \frac{\Delta_D^{1^+} - \Delta_D^l}{\Delta_{\text{step}}}, l = 1, 2, \cdots, L$, and the matrix elements in $(f_l + i, i), i = 1, 2, \cdots, n$, are 1, with all other matrix elements 0. Then, we define $\mathbf{T}_l := \mathbf{C}_l\mathbf{T}$. Using (2), the analyzer-free

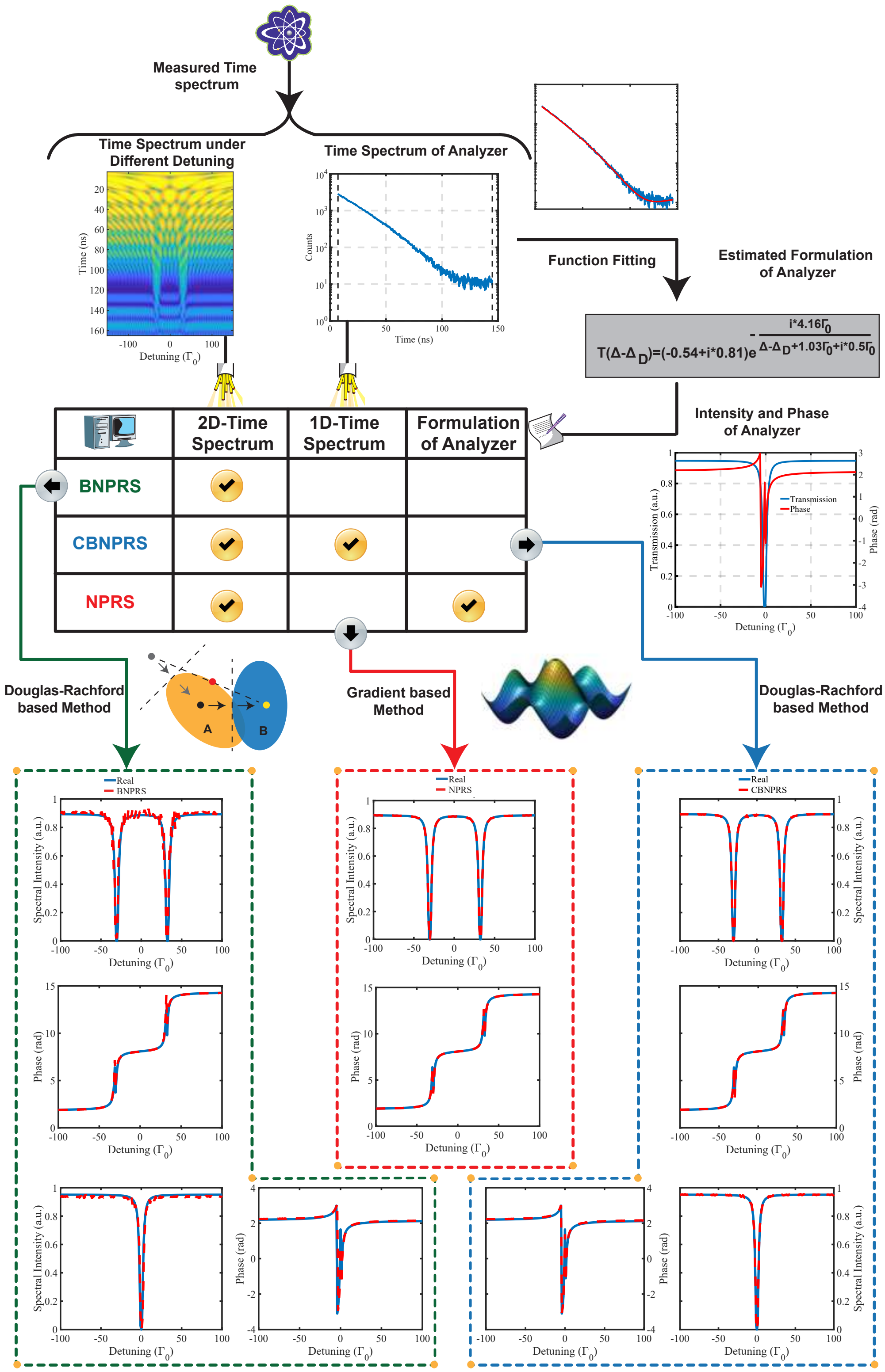

SUPPLEMENTARY FIG. 19. **Illustrative diagram presenting the differences and connections between NPRS, CB-NPRS and B-NPRS.** NPRS requires a model for the analyzer, whose parameters can be typically fitted from the measured time spectrum. B- and CB-NPRS do not require any analyzer model. CB-NPRS uses a measured time spectrum of the analyzer only as constraint.

model can be formulated as

$$\begin{aligned}&\text{Find } \mathbf{R}\in\mathbb{C}^{n},\mathbf{T}\in\mathbb{C}^{n+h}\\ &\text{s.t. } I(k,l)=\left|\mathbf{F}^{\mathrm{H}}_{:,k}\left(\mathbf{R}\odot(\mathbf{C}_l\mathbf{T})\right)\right|^2+\varepsilon,\\ &k=1,\cdots,K,\ l=1,\cdots,L.\end{aligned} \tag{12}$$

This formulation is actually a blind ptychography problem, which aims to recover the signal of interest $\mathbf{R}$ and the analyzer transmission $\mathbf{T}$ simultaneously. It is more challenging to deal with (12) than with (6).

Over the past decades, several methods have been proposed to solve the blind ptychography problem [18–21], see also Ref. [22] for a comprehensive review. Compared to the traditional blind ptychography problem in imaging, (12) has two obvious differences. First, in the traditional blind ptychography problem, $\mathbf{R}$ is divided into several local regions interacting with $\mathbf{T}$, which is not the case for (12). Second, for each l, $K\geq n$ in the traditional blind ptychography problem, while $K\ll n$ in (12), more specifically usually $K\approx 0.2n$. This indicates that (12) is more ill-conditioned than the traditional blind ptychography problem.

2. Analysis of the solution

Before solving (12), solutions of the problem should be analyzed starting with the noiseless cases with $\varepsilon=0$. As demonstrated in Refs. [23], there are two types of ambiguities inherent to (12). Specifically, if $\mathbf{R}_0$ and $\mathbf{T}_0$ are solutions of (12), then we have

- Scaling ambiguities: $c_1\mathbf{R}_0$, and $\frac{1}{c_1}\mathbf{T}_0$, are also solutions, with $c_1>0$.

- Affine phase ambiguities: $\mathbf{R}_0\odot e^{-i(\theta_1\mathbf{n}_1+\theta_2\mathbf{1}_n)}$, and $\mathbf{T}_0\odot e^{i(\theta_1\mathbf{n}_2-\theta_3\mathbf{1}_{n+h})}$, are also solutions, where $\mathbf{n}_1-\mathbf{C}_l\mathbf{n}_2=c_l\mathbf{1}_n$, $\mathbf{1}_n\in\mathbf{R}^n$ denotes the vector with all elements being 1, $\mathbf{n}_1\in\mathbb{R}^n,\mathbf{n}_2\in\mathbb{R}^{n+h}$, and $\theta_1,\theta_2,\theta_3,c_l\in\mathbf{R}$. For instance, $\mathbf{n}_1=(1,2,\cdots,n)$ and $\mathbf{n}_2=(1,2,\cdots,n+h)$.

Usually, the scaling ambiguities don't influence the analysis of Mössbauer spectrum, but the terms $\theta_1\mathbf{n}_1$ and $\theta_1\mathbf{n}_2$ in the affine phase ambiguities can change the relative phase of $\mathbf{R}_0$ and $\mathbf{T}_0$, which would accordingly destroy the information about the phase of $\mathbf{R}_0$ and $\mathbf{T}_0$. Fortunately, the intensities and phases of $\mathbf{R}_0$ and $\mathbf{T}_0$ remain nearly unchanged when $|\Delta|$

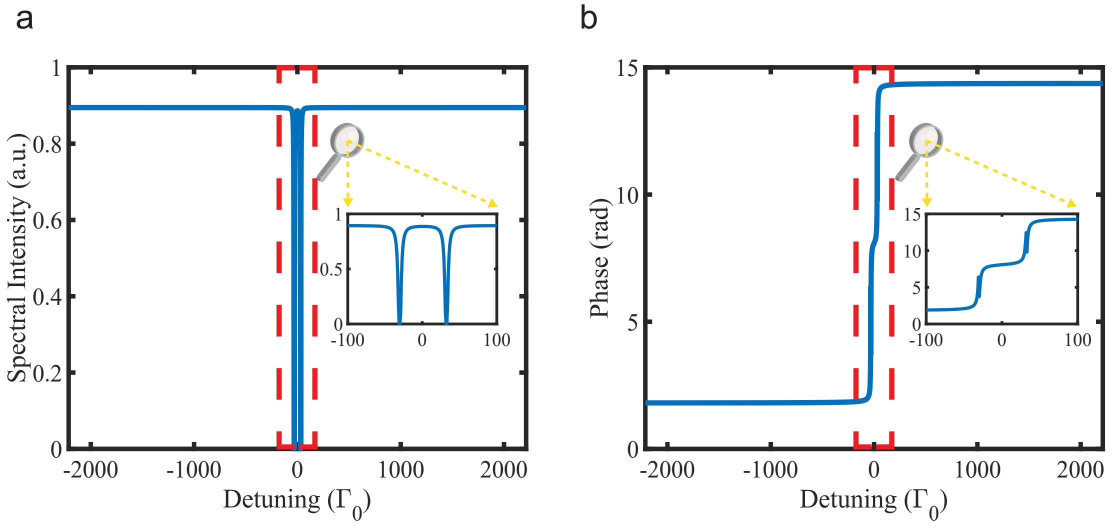

SUPPLEMENTARY FIG. 20. **Spectrum for large detuning ranges.** **a** The spectral intensity and **b** phase of the α-Fe sample of $2.3\,\mu$m with aligned magnetization and an analyzer of 1 μm effective thickness. When the detuning becomes large, the intensity and phase become nearly constant.

becomes large (for instance, $|\Delta| > 500\Gamma_0$), as shown in Fig. 20 for the α-Fe sample. This is due to the narrow resonance of the nuclear transitions. As a result, the variation of each element in $\theta_1\mathbf{n}_1$ and $\theta_1\mathbf{n}_2$ can be diminished, leading to the transformation of affine phase ambiguities into global phase ambiguities, specifically $\mathbf{R}_0 \odot e^{-i\theta_2\mathbf{1}_n}$ and $\mathbf{T}_0 \odot e^{-i\theta_3\mathbf{1}_{n+h}}$. This transformation ensures that the relative phases of both $\mathbf{R}_0$ and $\mathbf{T}_0$ remain unchanged.

Here, the B-NPRS algorithm implicitly employs this constraint by deliberately choosing the initialization, as detailed in Section K 3. Previous work in the literature has proven that the blind ptychography problem can have a unique solution except for these inherent ambiguities discussed above [20, 23]. However, in our case $K < n$ in (12) such that the number of measurement points are less than these theories require. Thus, similar to the conclusion in subsection B, we only guarantee $\mathbf{R}$ and $\mathbf{T}$ to be unique in the region of interest $\mathbf{O}$. As a result, in order to recover $\mathbf{R}$ well within the the region $\mathbf{O}$, the range of the Doppler detuning set $\boldsymbol{\Omega}$ should also cover $\mathbf{O}$ just like for the non-blind problem (6).

3. Introduction of algorithm

In this Section we introduce the B-NPRS algorithm to solve (12). The B-NPRS steps are shown in Algorithm 2.

Algorithm 2: Blind Nuclear Phase Retrieval Spectroscopy (B-NPRS)

Input:

$\mathbf{\Omega}$: the set of shifting detuning $\mathbf{\Omega} = \{\Delta_D^1, \Delta_D^2, \cdots, \Delta_D^L\}$

$\mathbf{E}$: the set of discrete time set $\mathbf{E} = \{t_1, t_2, \cdots, t_K\}$

$\mathbf{I} \in \mathbb{R}^{K \times L}$: the counts of photons

$\mathcal{A} := \left\{ \mathbf{\Psi} := (\mathbf{\Psi}_1, \mathbf{\Psi}_2, \ldots, \mathbf{\Psi}_L) \in \mathbb{C}^{n \times L} \mid \left| \mathbf{F}_{:,k}^{\mathrm{H}} \mathbf{\Psi}_l \right|^2 = I(k,l), 1 \le k \le K, 1 \le l \le L \right\}$

$\mathcal{B} :=$

$\left\{ \mathbf{\Psi} := (\mathbf{\Psi}_1, \mathbf{\Psi}_2, \ldots, \mathbf{\Psi}_L) \in \mathbb{C}^{n \times L} \,\middle|\, \exists\ \mathbf{R} \in \mathbb{C}^n, \mathbf{T} \in \mathbb{C}^{n+h},\ \text{s.t.} \mathbf{R} \odot (\mathbf{C}_l \mathbf{T}) = \mathbf{\Psi}_l, 1 \le l \le L \right\}$

τ: the error bound

Output:

$\hat{\mathbf{R}}$: an estimation of $\mathbf{R}$

$\hat{\mathbf{T}}$: an estimation of $\mathbf{T}$

Initialization:

$\mathbf{T}^{(0)} \in \mathbb{C}^{n+h}$ and $\mathbf{R}^{(0)} \in \mathbb{C}^n$ are the initializations

$\mathbf{\Psi}_l^{(0)} = \mathbf{R}^{(0)} \odot (\mathbf{C}_l \mathbf{T}^{(0)}),\ l = 1, \cdots, L$

General Step $(m = 1, 2, \cdots)$:

$\mathbf{\Psi}_l^{(m)_\mathcal{B}} = \mathbf{R}^{(m-1)} \odot (\mathbf{C}_l \mathbf{T}^{(m-1)}), l = 1, 2, \cdots, L$

$\mathbf{\Psi}_l^{(m)_{\mathcal{A}_{pre}}} = 2\mathbf{\Psi}_l^{(m)_\mathcal{B}} - \mathbf{\Psi}_l^{(m-1)}, l = 1, 2, \cdots, L$

$\mathbf{\Psi}^{(m)_\mathcal{A}} = \arg\min_{\mathbf{\Psi} \in \mathcal{A}} \sum_{l=1}^L \left\| \mathbf{\Psi}_l - \mathbf{\Psi}_l^{(m)_{\mathcal{A}_{pre}}} \right\|^2$

$\mathbf{\Psi}_l^{(m)} = \mathbf{\Psi}_l^{(m-1)} + \mathbf{\Psi}_l^{(m)_\mathcal{A}} - \mathbf{\Psi}_l^{(m)_\mathcal{B}}, l = 1, 2, \cdots, L$

$\mathbf{R}^{(m)} = \arg\min_{\mathbf{R}} \frac{1}{2} \sum_{l=1}^L \left\| \mathbf{\Psi}_l^{(m)} - \mathbf{R} \odot (\mathbf{C}_l \mathbf{T}^{(m-1)}) \right\|^2$

$\mathbf{T}^{(m)} = \arg\min_{\mathbf{T}} \frac{1}{2} \sum_{l=1}^L \left\| \mathbf{\Psi}_l^{(m-1)} - \mathbf{R}^{(m)} \odot (\mathbf{C}_l \mathbf{T}) \right\|^2$

if $\sum_{k=1}^K \sum_{l=1}^L \left(\left| \mathbf{F}_{:,k}^{\mathrm{H}} (\mathbf{R}^{(m)} \odot \left(\mathbf{C}_l \mathbf{T}^{(m)} \right)) \right|^2 - I(k,l) \right)^2 \le \tau$ **then**

$\hat{\mathbf{R}} = \mathbf{R}^{(m)}$

$\hat{\mathbf{T}} = \mathbf{T}^{(m)}$

Break

end if

The backbone of the B-NPRS algorithm is based on the Douglas Rachford (DR) method [16]. Given $\mathbf{\Psi} := (\mathbf{\Psi}_1, \mathbf{\Psi}_2, \ldots, \mathbf{\Psi}_L) \in \mathbb{C}^{n \times L}$, where $\mathbf{\Psi}_l := \mathbf{R} \odot (\mathbf{C}_l \mathbf{T})$, $1 \le l \le L$. (12) aims to find the $\mathbf{\Psi}^*$ which lies in the intersection of two sets namely $\mathbf{\Psi}^* \in \mathcal{A} \cap \mathcal{B}$, with

$$\mathcal{A} := \left\{ \mathbf{\Psi} := (\mathbf{\Psi}_1, \mathbf{\Psi}_2, \ldots, \mathbf{\Psi}_L) \in \mathbb{C}^{n \times L} \mid \left| \mathbf{F}_{:,k}^{\mathrm{H}} \mathbf{\Psi}_l \right|^2 = I(k,l), 1 \le k \le K, 1 \le l \le L \right\},$$

$$\mathcal{B} := \left\{ \mathbf{\Psi} := (\mathbf{\Psi}_1, \mathbf{\Psi}_2, \ldots, \mathbf{\Psi}_L) \in \mathbb{C}^{n \times L} \,\middle|\, \exists\ \mathbf{R} \in \mathbb{C}^n, \mathbf{T} \in \mathbb{C}^{n+h},\ \text{s.t.} \mathbf{R} \odot (\mathbf{C}_l \mathbf{T}) = \mathbf{\Psi}_l, 1 \le l \le L \right\}.$$

Then, $\hat{\mathbf{R}}$ and $\hat{\mathbf{T}}$ are calculated by

$$\left(\hat{\mathbf{R}}, \hat{\mathbf{T}}\right) = \arg\min_{\mathbf{R},\mathbf{T}} \frac{1}{2} \sum_{l=1}^{L} \|\mathbf{\Psi}_l^* - \mathbf{R} \odot (\mathbf{C}_l \mathbf{T})\|^2 .$$

At iteration m, DR method iteratively reflects $\mathbf{\Psi}^{(m-1)}$ between $\mathcal{A}$ and $\mathcal{B}$ to achieve

$$\begin{aligned}\tilde{\mathbf{\Psi}}^{(m)} &= (2\mathbb{P}_{\mathcal{A}} - \mathbb{I})(2\mathbb{P}_{\mathcal{B}} - \mathbb{I})(\mathbf{\Psi}^{(m-1)}) \\ &= 2\mathbb{P}_{\mathcal{A}}(2\mathbb{P}_{\mathcal{B}} - \mathbb{I})(\mathbf{\Psi}^{(m-1)}) - (2\mathbb{P}_{\mathcal{B}} - \mathbb{I})(\mathbf{\Psi}^{(m-1)}),\end{aligned}$$

where $\mathbb{P}_{\mathcal{A}}(\cdot)$ and $\mathbb{P}_{\mathcal{B}}(\cdot)$ are the projectors of $\mathcal{A}$ and $\mathcal{B}$. Finally, we make an average between $\mathbf{\Psi}^{(m-1)}$ and $\tilde{\mathbf{\Psi}}^{(m)}$ to obtain $\mathbf{\Psi}^{(m)}$ namely

$$\begin{aligned}\mathbf{\Psi}^{(m)} &= \frac{1}{2}(\mathbf{\Psi}^{(m-1)} + \tilde{\mathbf{\Psi}}^{(m)}) \\ &= \mathbf{\Psi}^{(m-1)} + \mathbb{P}_{\mathcal{A}}\left(2\mathbb{P}_{\mathcal{B}}(\mathbf{\Psi}^{(m-1)}) - \mathbf{\Psi}^{(m-1)}\right) - \mathbb{P}_{\mathcal{B}}(\mathbf{\Psi}^{(m-1)}).\end{aligned}$$

Let $\mathbf{\Psi}^{(m)_{\mathcal{A}}} := \mathbb{P}_{\mathcal{A}}(\mathbf{\Psi}^{(m-1)})$, which can be estimated by solving the problem

$$\mathbb{P}_{\mathcal{A}}(\mathbf{\Psi}^{(m-1)}) = \arg\min_{\mathbf{\Psi}\in\mathcal{A}} \sum_{l=1}^{L} \left\|\mathbf{\Psi}_l - \mathbf{\Psi}_l^{(m-1)}\right\|^2 .$$

More specifically, if $t_1, t_2, \cdots, t_K$ are multiples of t_{step}, and $n = \lfloor \frac{2\pi}{t_{\text{step}}\Delta_{\text{step}}} \rfloor + 1$ where $\lfloor\cdot\rfloor$ denotes rounding down, we have

$$\mathbf{F}_{:,k} = \frac{\Delta_{\text{step}}}{\sqrt{2\pi}}(1, e^{i\Delta_{\text{step}}\cdot t_k}, \cdots, e^{i(n-1)\Delta_{\text{step}}\cdot t_k}) \approx \frac{\Delta_{\text{step}}}{\sqrt{2\pi}}(1, e^{\frac{2\pi i \lfloor \frac{t_k}{t_{\text{step}}} \rfloor}{n}}, \cdots, e^{\frac{2\pi i(n-1)\lfloor \frac{t_k}{t_{\text{step}}} \rfloor}{n}}).$$

Next, define $\mathbf{F}_{n\times n}$ to be a standard Fourier matrix. Then, the $\lfloor \frac{t_k}{t_{\text{step}}} \rfloor$th row of $\frac{\Delta_{\text{step}}}{\sqrt{2\pi}}\mathbf{F}_{n\times n}$ is $\mathbf{F}_{:,k}^{\text{H}}$, where $(\cdot)^{\text{H}}$ denotes the conjugate transpose. Next, let $\hat{\mathbf{\Psi}}_l^{(m)} = \frac{\Delta_{\text{step}}}{\sqrt{2\pi}}\mathbf{F}_{n\times n}\mathbf{\Psi}_l^{(m-1)}, l = 1, 2, \cdots, L$. Introducing $\mathcal{I} = \{\lfloor \frac{t_1}{t_{\text{step}}} \rfloor, \lfloor \frac{t_2}{t_{\text{step}}} \rfloor, \cdots, \lfloor \frac{t_K}{t_{\text{step}}} \rfloor\}$, we modify $\hat{\mathbf{\Psi}}_l^{(m)}$ as

$$\hat{\Psi}_l^{(m)}(k) = \begin{cases} \sqrt{I_l(k)} \odot \dfrac{\hat{\Psi}_l^{(m)}(k)}{\left|\hat{\Psi}_l^{(m)}(k)\right|}, k \in \mathcal{I} \\ \hat{\Psi}_l^{(m)}(k), \text{else} \end{cases}, \ l = 1, 2, \cdots, L.$$

Finally, we can estimate $\mathbb{P}_{\mathcal{A}}(\mathbf{\Psi}^{(m-1)})$ as

$$\mathbb{P}_{\mathcal{A}}(\mathbf{\Psi}^{(m-1)}) = \left\{ \frac{\sqrt{2\pi}}{\Delta_{\text{step}}} \mathbf{F}_{n\times n}^{-1} \hat{\mathbf{\Psi}}_1^{(m)}, \frac{\sqrt{2\pi}}{\Delta_{\text{step}}} \mathbf{F}_{n\times n}^{-1} \hat{\mathbf{\Psi}}_2^{(m)}, \cdots, \frac{\sqrt{2\pi}}{\Delta_{\text{step}}} \mathbf{F}_{n\times n}^{-1} \hat{\mathbf{\Psi}}_L^{(m)} \right\}.$$

The projection $\mathbf{\Psi}^{(m)_{\mathcal{B}}} := \mathbb{P}_{\mathcal{B}}(\mathbf{\Psi}^{(m-1)}), m \geq 2$ can be calculated by the two steps

$$i) \quad \left(\mathbf{R}^{(m-1)}, \mathbf{T}^{(m-1)}\right) = \arg\min_{\mathbf{R},\mathbf{T}} \frac{1}{2} \sum_{l=1}^{L} \left\| \mathbf{\Psi}_l^{(m-1)} - \mathbf{R} \odot (\mathbf{C}_l \mathbf{T}) \right\|^2.$$

$$ii) \quad \mathbf{\Psi}_l^{(m)_{\mathcal{B}}} = \mathbf{R}^{(m-1)} \odot (\mathbf{C}_l \mathbf{T}^{(m-1)}), l = 1, 2, \cdots, L.$$

In practice, $i)$ can be solved inexactly by the alternating minimization as

$$\begin{aligned} \mathbf{R}^{(m-1)} &= \arg\min_{\mathbf{R}\in\mathbb{C}^n} \frac{1}{2} \sum_{l=1}^{L} \left\| \mathbf{\Psi}_l^{(m-1)} - \mathbf{R} \odot (\mathbf{C}_l \mathbf{T}^{(m-2)}) \right\|^2 \\ &= \frac{\sum_{l=1}^{L} \overline{(\mathbf{C}_l \mathbf{T}^{(m-2)})} \odot \mathbf{\Psi}_l^{(m-1)}}{\sum_{l=1}^{L} |\mathbf{C}_l \mathbf{T}^{(m-2)}|^2}, \end{aligned}$$

where $\overline{\cdot}$, $|\cdot|^2$, and $\dot{\overline{\cdot}}$ are the element-wise operators, and

$$\mathbf{T}^{(m-1)} = \arg\min_{\mathbf{T}\in\mathbb{C}^{n+h}} \frac{1}{2} \sum_{l=1}^{L} \left\| \mathbf{\Psi}_l^{(m-1)} - \mathbf{R}^{(m-1)} \odot (\mathbf{C}_l \mathbf{T}) \right\|^2. \tag{13}$$

If the matrix $\sum_{l=1}^{L} \mathbf{C}_l^{\text{H}} \text{diag}(|\mathbf{R}^{(m-1)}|^2)_{n\times n} \mathbf{C}_l$ is invertible, the solution of (13) has a closed form

$$\mathbf{T}^{(m-1)} = \left(\sum_{l=1}^{L} \mathbf{C}_l^{\text{H}} \text{diag}\left(|\mathbf{R}^{(m-1)}|^2\right)_{n\times n} \mathbf{C}_l \right)^{-1} \left(\sum_{l=1}^{L} \mathbf{C}_l^{\text{H}} \left(\overline{\mathbf{R}^{(m-1)}} \odot \mathbf{\Psi}_l^{(m-1)} \right) \right),$$

where $\text{diag}(\cdot)_{n\times n}$ is to transform a length n vector into a $n \times n$ diagonal matrix. Else, the gradient descent algorithm can be applied to estimate the solution of (13).

In the following, we give some further technical details such as how to choose a proper initialization and how to make the B-NPRS method be robust the noise.

Initialization: Choosing the initialization is important for achieving a good result when dealing with a non-convex problem like (12). At the same time, as discussed in Sec. K 2, the solution of (12) has the affine phase ambiguities which can destroy the structure of the

phase of the ground truth $\mathbf{R}$. In order to relieve the influence caused by the affine phase ambiguities, the initializations $\mathbf{R}^{(0)}$ and $\mathbf{T}^{(0)}$ should have the property that they change slowly when $|\Delta|$ is large. So the initialization for $\mathbf{R}^{(0)}$ is also chosen as $\alpha\mathbf{1}, \alpha \in (0,1)$, as in the NPRS algorithm. To avoid $\left|\mathbf{F}^{\mathrm{H}}_{:,k}\left(\mathbf{R}^{(0)} \odot (\mathbf{C}_l\mathbf{T}^{(0)})\right)\right|^2 = 0$ for any k and l, the initialization for $\mathbf{T}^{(0)}$ is chosen as a piece-wise constant vector namely

$$\mathbf{T}^{(0)}(\Delta) = \begin{cases} \alpha_1, \Delta \in \mathbf{O} \\ \alpha_2, \text{else} \end{cases}, \tag{14}$$

where $\alpha_1, \alpha_2 \in (0,1)$ are two different constants, and $\mathbf{O}$ is the region of interest. On the other hand, we can also choose a roughly known analyzer function as the initialization $\mathbf{T}^{(0)}$.

Robustness: The performance of a DR-based algorithm like B-NPRS is liable to get deteriorated when the data sets are corrupted with noise. Thus, as suggested in Ref. [22], we also perform a weighted average with a tunable parameter $\beta \in (0,1)$ to calculate $\mathbf{\Psi}_l^{(m)}$ as

$$\mathbf{\Psi}_l^{(m)} = \beta\left(\mathbf{\Psi}_l^{(m-1)} + \mathbf{\Psi}_l^{(m)\mathcal{A}} - \mathbf{\Psi}_l^{(m)\mathcal{B}}\right) + (1-\beta)\mathbf{\Psi}_l^{(m)\mathcal{B}}, l = 1, 2, \cdots, L. \tag{15}$$

When dealing with experimental data, we often choose $\beta \in (0.85, 1)$ to improve the performance of the B-NPRS algorithm.

Next, we will apply the B-NPRS algorithm to the four numerical simulations considered so far and to the nuclear forward scattering experimental data set.

4. Numerical simulations

We test the ability of the B-NPRS algorithm using the four cases discussed in previous sections. The initialization of $\mathbf{R}^{(0)}$ is $\alpha\mathbf{1}, \alpha > 0$, and the initialization $\mathbf{T}^{(0)}$ is the piece-wise constant vector shown in (14). At each iteration, we also record the measurement error and relative error of $\hat{\mathbf{R}}$ and $\hat{\mathbf{T}}$ respectively. As presented in Figs. 21-24, the B-NPRS algorithm works well for all four cases, successfully recovering both the intensity and phase of the target and the analyzer. Compared to the NPRS algorithm, the B-NPRS results show a slightly larger measured error and relative error of the target, however without requiring any prior information about the analyzer. The recovered phase is accurate, including for instance also the phase jumps at approx. $\pm 30\Gamma_0$ for the case of the nuclear forwards scattering setup with

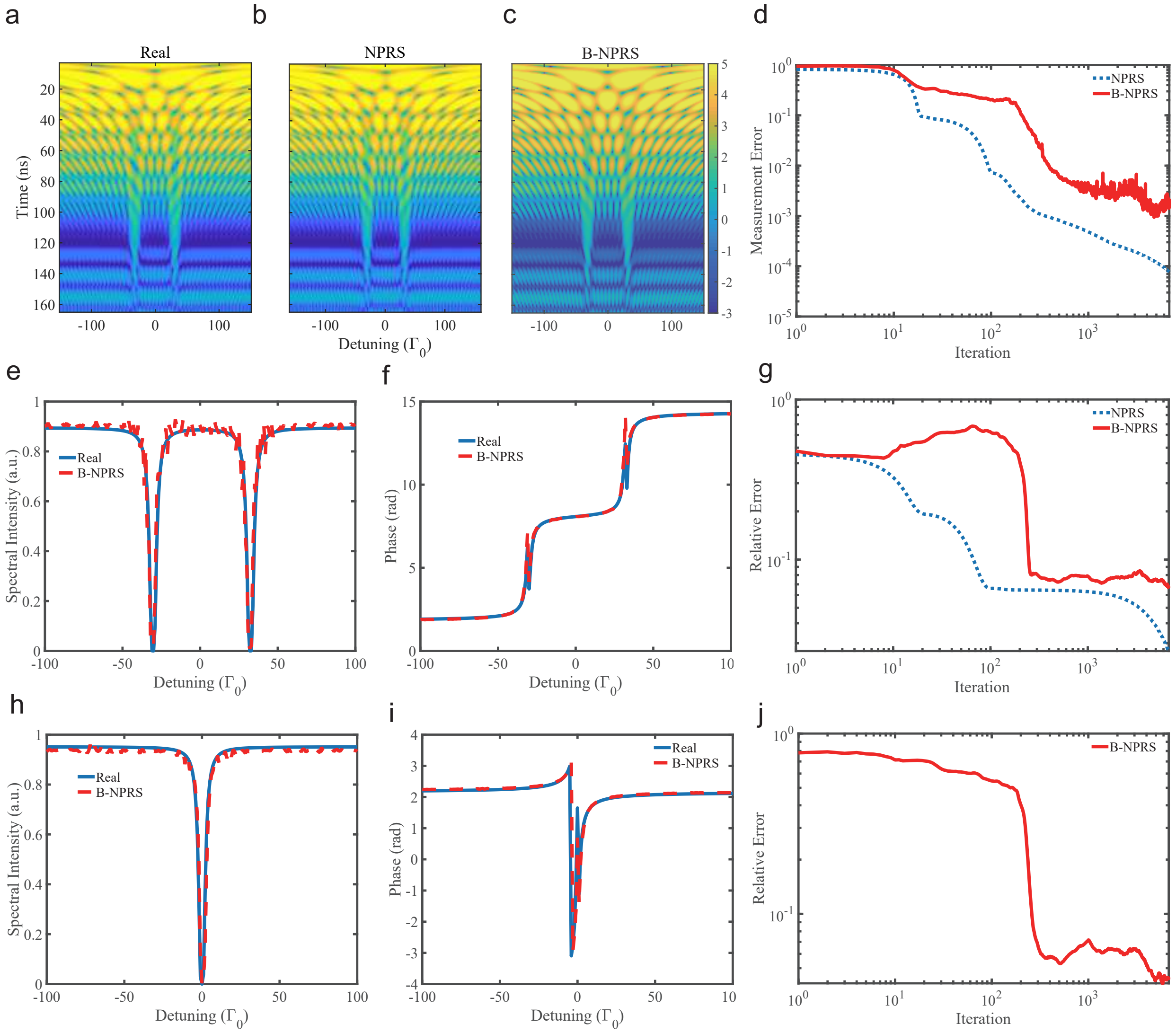

SUPPLEMENTARY FIG. 21. **Numerical results for the α-Fe sample of $2.3\,\mu$m with aligned magnetization and an analyzer of 1 μm effective thickness. a-c** are the real 2D spectrum, and 2D spectrum recovered by the NPRS and B-NPRS algorithms, respectively. **d** records the measurement error of the NPRS and B-NPRS algorithms. **e-f**, and **h-i** show the recovered spectral intensities and phases of the target ($\mathbf{R}$) and the analyzer ($\mathbf{T}$). **g** and **j** record the relative errors of the target and the analyzer.

aligned magnetization illustrated in Fig. 21.

5. *Experimental results*

We now apply the B-NPRS algorithm to the experimental data set for an α-Fe sample with aligned magnetization presented in the main text. The results including the reconstructed

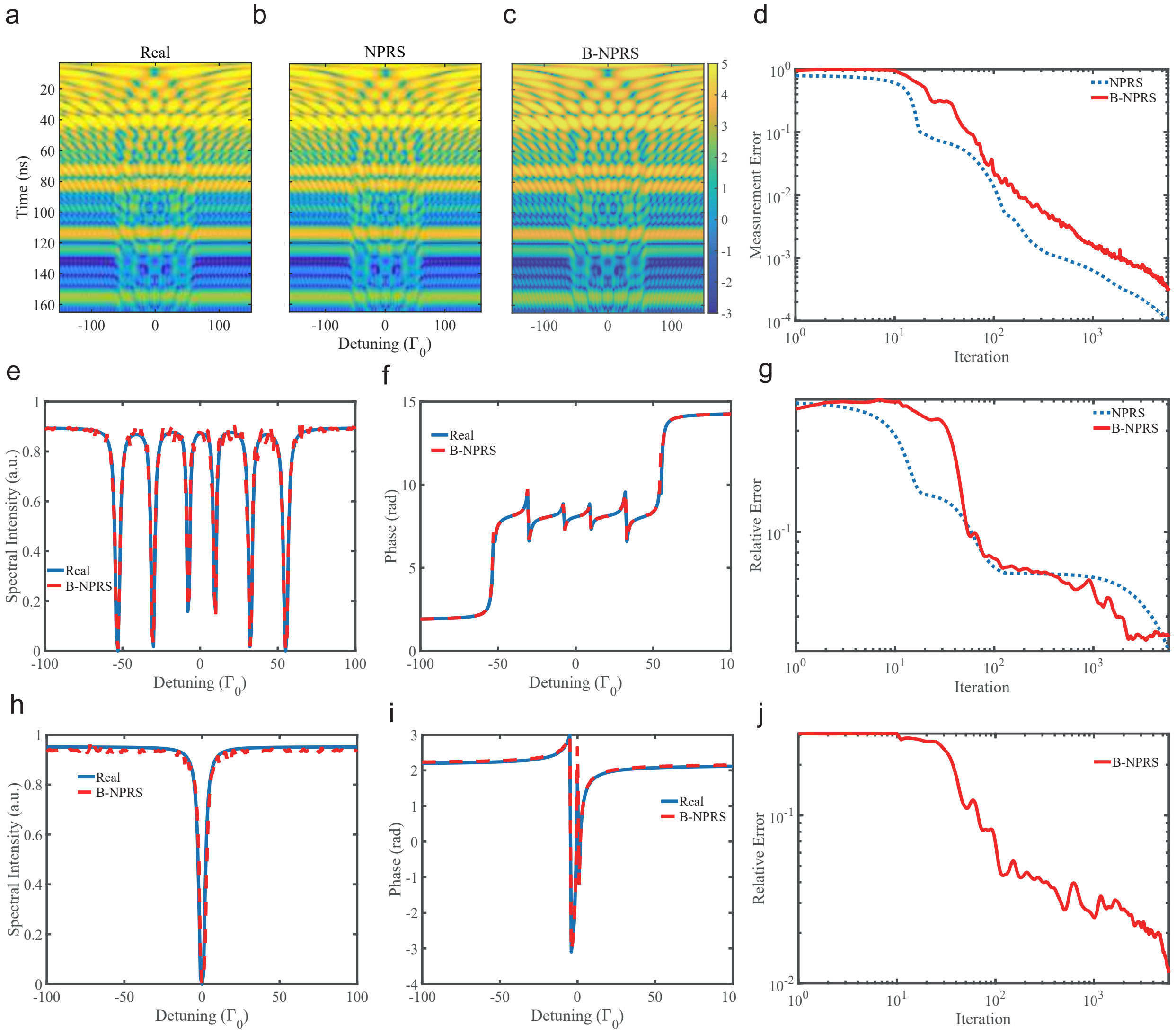

SUPPLEMENTARY FIG. 22. **Numerical results for an α-Fe sample of 2.3 μm with random magnetization.** **a-c** are the real 2D spectrum, and 2D spectrum recovered by the NPRS and B-NPRS algorithms, respectively. **d** records the measurement error of NPRS and B-NPRS. **e-f**, and **h-i** show the recovered spectral intensities and phases of the target (**R**) and the analyzer (**T**). **g** and **j** record the relative errors of the target and the analyzer.

energy and phase spectra and the measurement error are presented in Fig. 25. Unlike the numerical case presented in Fig. 21, the B-NPRS algorithm has a lower measurement error compared to the NPRS one, as shown in Fig. 25 **d**. This is due to noise, which appears in the experimental data and is not present for the numerical simulations. The B-NPRS algorithm, which has no constrains regarding the analyzer function, tends to overfit the noise. Thus, the B-NPRS measurement error drops faster, however without providing a more accurate retrieval of the energy spectrum, which presents more baseline oscillations than the NPRS

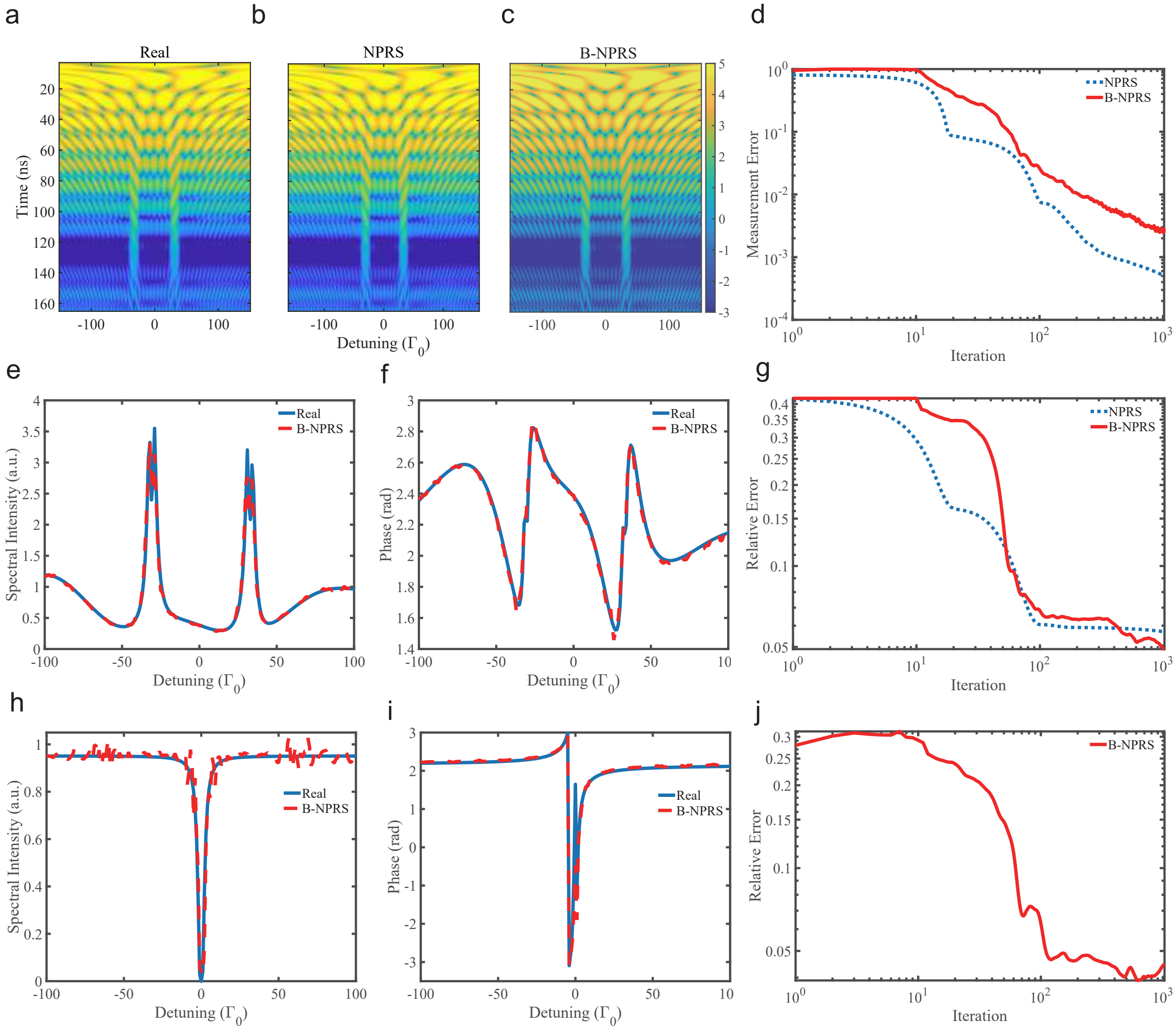

SUPPLEMENTARY FIG. 23. **Numerical results for the case of fast mechanical motion with the displacement** 0.43 **Å applied on an** α**-Fe sample of** $2.0\,\mu$**m with aligned magnetization. a-c** are the real 2D spectrum, and 2D spectrum recovered by the NPRS and B-NPRS algorithms, respectively. **d** records the measurement error of NPRS and B-NPRS. **e-f**, and **h-i** show the recovered spectral intensities and phases of the target (**R**) and the analyzer (**T**). **g** and **j** record the relative errors of the target and the analyzer.

result. The overall measurement error is of course larger than for the numerical simulation case presented in Fig. 21. Just as with the NPRS algorithm, the recovered phase misses the phase jumps at approx. $\pm 30\Gamma_0$, which are accessible for the numerical data set, but elusive for the experimental data set.

The performance of the B-NPRS and NPRS algorithms can be verified by an independent cross-check with the target's measured time spectrum, as shown in Fig. 25 **g**. The time

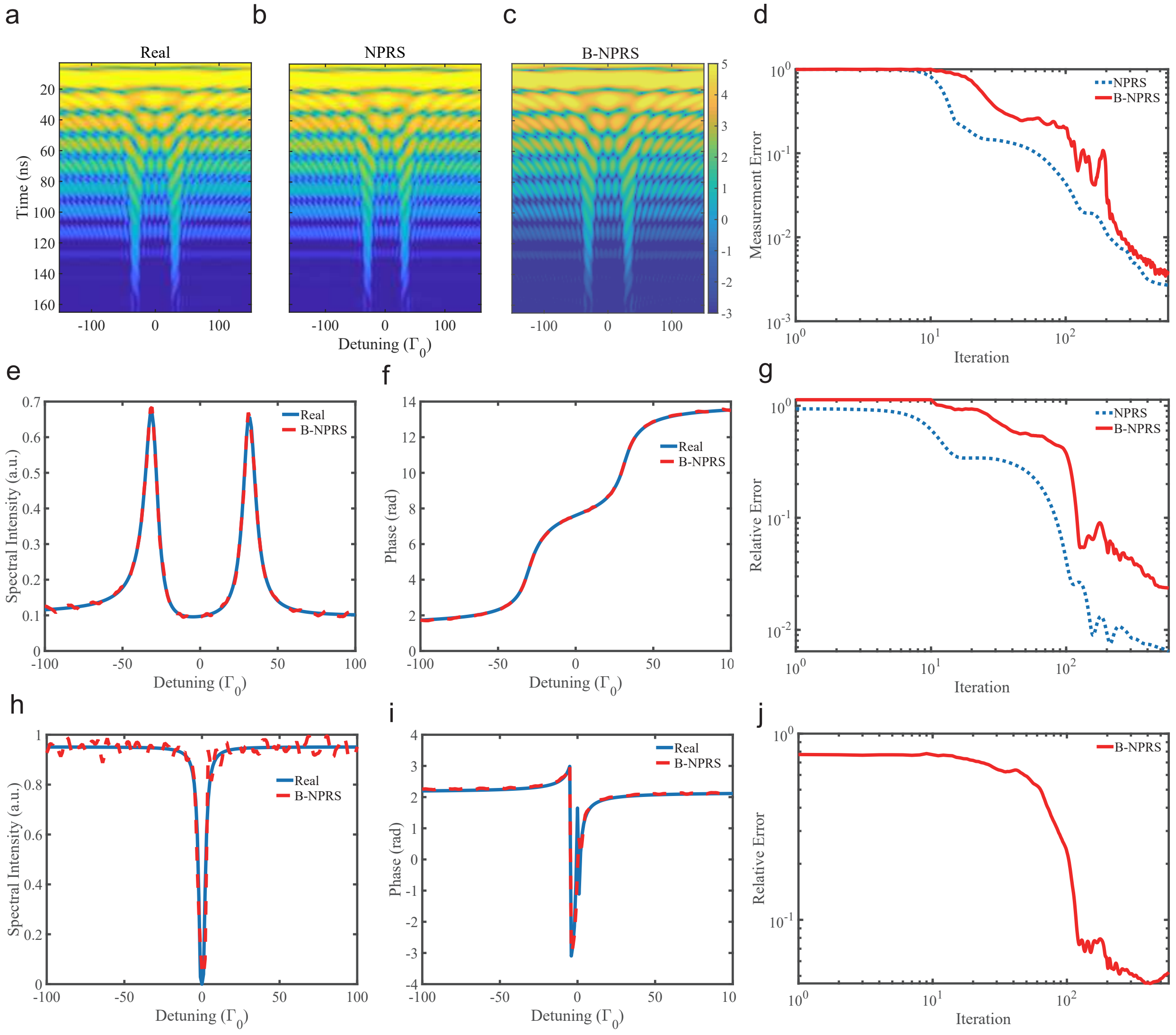

SUPPLEMENTARY FIG. 24. **Numerical results for a thin-film cavity with the structure Ta (2 nm)/B (17 nm)/**^{57}Fe**(3 nm)/B (7 nm)/Ta (9 nm) at the incidence angle** 3.5 **mrad.** **a-c** are the real 2D spectrum, and 2D spectrum recovered by the NPRS and B-NPRS algorithms, respectively. **d** records the measurement error of NPRS and B-NPRS. **e-f**, and **h-i** show the recovered spectral intensities and phases of the target (**R**) and the analyzer (**T**). **g** and **j** record the relative errors of the target and the analyzer.

spectra of both target and analyzer (for which a measured spectrum was taken) have been reconstructed using a numerical Fourier transform, as both spectra are originally recovered by B-NPRS in the frequency domain. The NPRS time spectrum fits the experimental data better than that of the B-NPRS algorithm. The intensity and phase of the analyzer have been successfully recovered using the B-NPRS algorithm, as shown in Fig. 25 **h** and **i**, and verified by an independent cross-check with the analyzer's measured time spectrum, as

presented in Fig. 25 **j**. Especially for large times, the B-NPRS time spectra for both target and analyzer present far more noise features than the NPRS ones. Nevertheless, given the fact that B-NPRS does not require any information on the analyzer, the results are still suprisingly good. However, if a reliable model of the analyzer exists, NPRS spectra present superior accuracy.

L. Constrained Blind Nuclear Phase Retrieval Spectroscopy

1. Introduction of algorithm

In experiments, often a time spectrum of the analyzer alone $\mathbf{I_T}$ is available, i.e., such a spectrum can be measured. Thus, without considering any theoretical model for the analyzer, we could use such a measured spectrum of the analyzer alone to constrain the feasible space and relieve the ill-conditioned nature of (12). Just like (12), we also assume $\Delta_D^1, \Delta_D^2, \cdots, \Delta_D^L$, which are the integer multiples of Δ_{step}, are ordered decreasingly, and for each Δ_D^l, the lth column of $\mathbf{I}$ and $\mathbf{I_T}$ has the same number of sampling points. Then the model combining the constraints of $\mathbf{I_T}$ can be formulated as

$$\begin{aligned} &\text{Find } \mathbf{R} \in \mathbb{C}^n, \mathbf{T} \in \mathbb{C}^{n+h} \\ &\text{s.t. } I(k,l) = \left|\mathbf{F}_{:,k}^{\text{H}} \left(\mathbf{R} \odot (\mathbf{C}_l \mathbf{T})\right)\right|^2 + \varepsilon_1, \\ &k = 1, \cdots, K, \; l = 1, \cdots, L, \\ &I_{\mathbf{T}}(k) = \left|\mathbf{F}_{:,k}^{\text{H}} \mathbf{C}_{L+1} \mathbf{T}\right|^2 + \varepsilon_2, \; k = 1, \cdots, K\,, \end{aligned} \tag{16}$$

where ε_1 and ε_2 are noise, and

$$\mathbf{C}_{L+1} = \begin{array}{c} \\ {\color{blue}1} \\ {\color{blue}2} \\ {\color{blue}\vdots} \\ {\color{blue}n} \end{array} \begin{array}{c} \begin{array}{ccccccccc} {\color{blue}1} & {\color{blue}2} & {\color{blue}\cdots} & {\color{blue}f_{L+1}+1} & {\color{blue}f_{L+1}+2} & {\color{blue}\cdots} & {\color{blue}f_{L+1}+n} & {\color{blue}\cdots} & {\color{blue}h+n} \end{array} \\ \begin{pmatrix} 0 & 0 & \cdots & 1 & 0 & \cdots & 0 & \cdots & 0 \\ 0 & 0 & \cdots & 0 & 1 & \cdots & 0 & \cdots & 0 \\ \vdots & \vdots & \cdots & \vdots & \vdots & \ddots & \vdots & \cdots & \vdots \\ 0 & 0 & \cdots & 0 & 0 & \cdots & 1 & \cdots & 0\,, \end{pmatrix} \end{array}$$

where $f_{L+1} = \frac{\Delta_D^{1^+}}{\Delta_{\text{step}}}$ and once more the matrix row and column indices are indicated in blue.

Compared to (12), (16) can naturally mitigate the influence caused by the affine phase

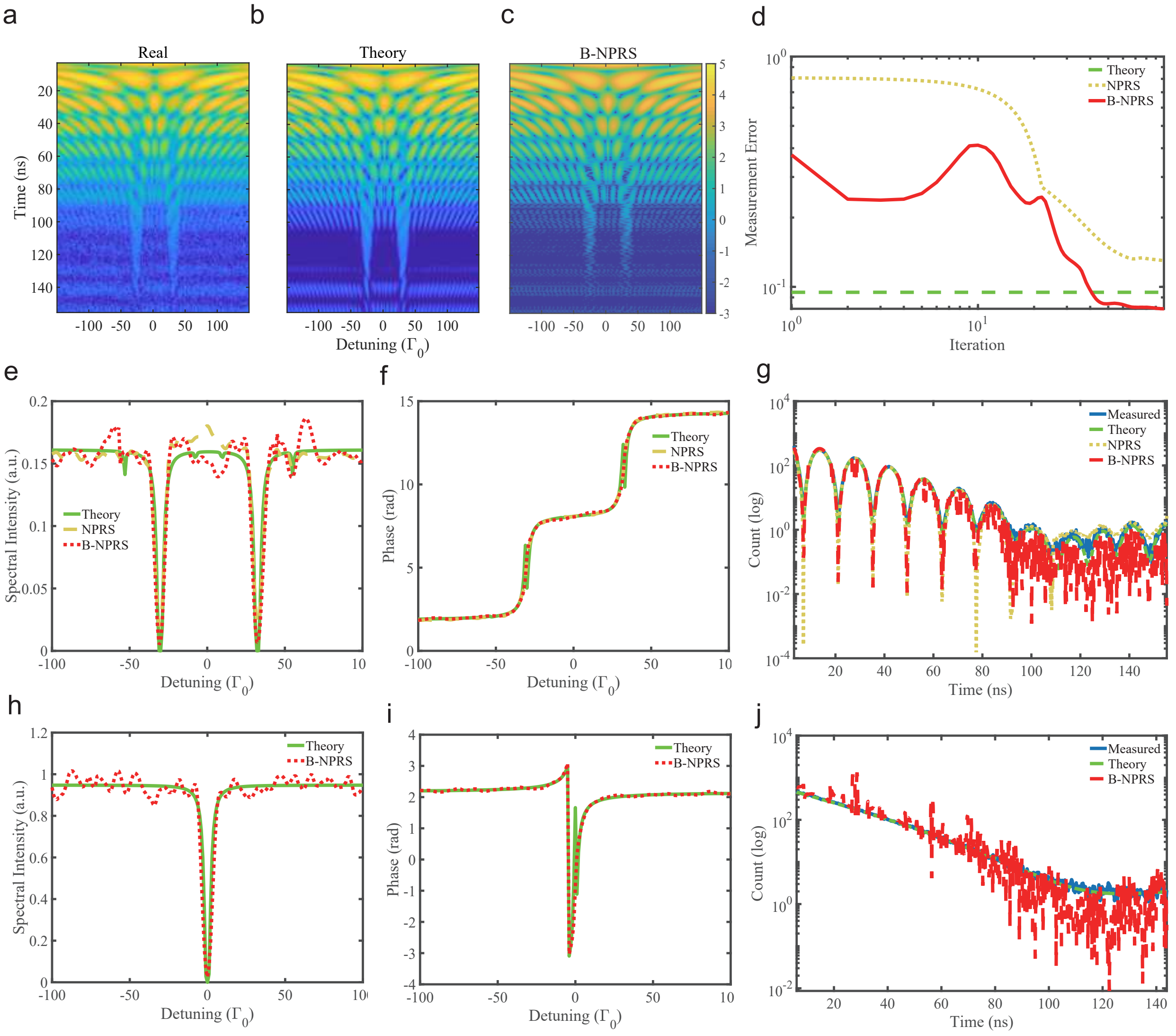

SUPPLEMENTARY FIG. 25. **Experimental results for α-Fe sample of $2.3\,\mu$m with an aligned magnetization and an analyzer of 1 μm effective thickness.** **a-c** present the real 2D spectrum, and 2D spectra recovered by the NPRS and B-NPRS algorithms, respectively. **d** records the measurement error of NPRS and B-NPRS. **e-f**, and **h-i** show the recovered spectral intensities and phases of the target ($\mathbf{R}$) and the analyzer ($\mathbf{T}$). **g** and **j** plot the recovered and measured time spectra of target and analyzer, respectively. A comparison is made with theory modeling which extracts the two response functions from measured time spectra of target and analyzer.

ambiguity with the aid of $\mathbf{I_T}$. This property improves the robustness of (16) over (12). Consequently, we develop a Constrained Blind Nuclear Phase Retrieval Spectroscopy (CB-NPRS) algorithm that preserves the time spectrum of the analyzer to solve (16). The CB-NPRS steps are presented in Algorithm 3.

Algorithm 3: Constrained Blind Nuclear Phase Retrieval Spectroscopy (CB-NPRS)

Input:

$\mathbf{\Omega}$: the set of shifting detuning $\mathbf{\Omega} = \{\Delta_D^1, \Delta_D^2, \cdots, \Delta_D^L\}$

$\mathbf{E}$: the set of discrete time set $\mathbf{E} = \{t_1, t_2, \cdots, t_K\}$

$\mathbf{I} \in \mathbb{R}^{K \times L}$: the counts of photons for $\mathbf{R}$ and $\mathbf{T}$.

$\mathbf{I_T} \in \mathbb{R}^{K}$: the counts of photons for $\mathbf{T}$.

$$\mathcal{A} := \Big\{ \mathbf{\Psi} := (\mathbf{\Psi}_1, \mathbf{\Psi}_2, \ldots, \mathbf{\Psi}_L, \mathbf{\Psi}_{L+1}) \in \mathbb{C}^{n \times (L+1)} \;\Big|\; \left|\mathbf{F}^{\mathrm{H}}_{:,k} \mathbf{\Psi}_l\right|^2 = I(k,l), 1 \le k \le K, 1 \le l \le L\,, \\ \left|\mathbf{F}^{\mathrm{H}}_{:,k} \mathbf{\Psi}_{L+1}\right|^2 = I_{\mathbf{T}}(k), 1 \le k \le K \Big\}.$$

$$\mathcal{B} := \{ \mathbf{\Psi} := (\mathbf{\Psi}_1, \mathbf{\Psi}_2, \ldots, \mathbf{\Psi}_L, \mathbf{\Psi}_{L+1}) \in \mathbb{C}^{n \times (L+1)} \;|\exists\; \mathbf{R} \in \mathbb{C}^n, \mathbf{T} \in \mathbb{C}^{n+h}, \text{ s.t.} \mathbf{C}_{L+1}\mathbf{T} = \mathbf{\Psi}_{L+1}\,, \\ \mathbf{R} \odot (\mathbf{C}_l \mathbf{T}) = \mathbf{\Psi}_l, 1 \le l \le L \}.$$

τ: the error bound

λ: the parameter

Output:

$\hat{\mathbf{R}}$: an estimation of $\mathbf{R}$

$\hat{\mathbf{T}}$: an estimation of $\mathbf{T}$

Initialization:

$\mathbf{T}^{(0)} \in \mathbb{C}^{n+h}$ and $\mathbf{R}^{(0)} \in \mathbb{C}^n$ are the initializations

$\mathbf{\Psi}_l^{(0)} = \mathbf{R}^{(0)} \odot (\mathbf{C}_l \mathbf{T}^{(0)}),\ l = 1, \cdots, L, \mathbf{\Psi}_{L+1}^{(0)} = \mathbf{C}_{L+1}\mathbf{T}^{(0)}$

General Step $(m = 1, 2, \cdots)$:

$\mathbf{\Psi}_l^{(m)_\mathcal{B}} = \mathbf{R}^{(m-1)} \odot (\mathbf{C}_l \mathbf{T}^{(m-1)}), l = 1, 2, \cdots, L, \mathbf{\Psi}_{L+1}^{(m)_\mathcal{B}} = \mathbf{C}_{L+1}\mathbf{T}^{(m-1)}$

$\mathbf{\Psi}_l^{(m)_{\mathcal{A}_{pre}}} = 2\mathbf{\Psi}_l^{(m)_\mathcal{B}} - \mathbf{\Psi}_l^{(m-1)}, l = 1, 2, \cdots, L+1$

$\mathbf{\Psi}^{(m)_\mathcal{A}} = \arg\min_{\mathbf{\Psi} \in \mathcal{A}} \sum_{l=1}^{L+1} \left\| \mathbf{\Psi}_l - \mathbf{\Psi}_l^{(m)_{\mathcal{A}_{pre}}} \right\|^2$

$\mathbf{\Psi}_l^{(m)} = \mathbf{\Psi}_l^{(m-1)} + \mathbf{\Psi}_l^{(m)_\mathcal{A}} - \mathbf{\Psi}_l^{(m)_\mathcal{B}}, l = 1, 2, \cdots, L+1$

$\mathbf{R}^{(m)} = \arg\min_{\mathbf{R}} \frac{1}{2} \sum_{l=1}^{L} \left\| \mathbf{\Psi}_l^{(m)} - \mathbf{R} \odot (\mathbf{C}_l \mathbf{T}^{(m-1)}) \right\|^2$

$\mathbf{T}^{(m)} = \arg\min_{\mathbf{T}} \frac{1}{2} \sum_{l=1}^{L} \left\| \mathbf{\Psi}_l^{(m)} - \mathbf{R}^{(m)} \odot (\mathbf{C}_l \mathbf{T}) \right\|^2 + \frac{\lambda}{2} \left\| \mathbf{\Psi}_{L+1}^{(m)} - \mathbf{C}_{L+1}\mathbf{T} \right\|^2$

if

$\sum_{k=1}^{K} \left(\sum_{l=1}^{L} \left(\left| \mathbf{F}^{\mathrm{H}}_{:,k} (\mathbf{R}^{(m)} \odot (\mathbf{C}_l \mathbf{T}^{(m)})) \right|^2 - I(k,l) \right)^2 + \left(\left| \mathbf{F}^{\mathrm{H}}_{:,k} \mathbf{C}_{L+1} \mathbf{T}^{(m)} \right|^2 - I_{\mathbf{T}}(k) \right)^2 \right) \le \tau$

then

$\hat{\mathbf{R}} = \mathbf{R}^{(m)}$

$\hat{\mathbf{T}} = \mathbf{T}^{(m)}$

Break

end if

The CB-NPRS and B-NPRS algorithms are almost the same, except for the update of $\mathbf{T}^{(m)}$ as below

$$\mathbf{T}^{(m)} = \arg\min_{\mathbf{T}} \frac{1}{2} \sum_{l=1}^{L} \left\| \mathbf{\Psi}_l^{(m)} - \mathbf{R}^{(m)} \odot (\mathbf{C}_l \mathbf{T}) \right\|^2 + \frac{\lambda}{2} \left\| \mathbf{\Psi}_{L+1}^{(m)} - \mathbf{C}_{L+1}\mathbf{T} \right\|^2, \tag{17}$$

where $\lambda > 0$ is the parameter to control the constraints by the analyzer time spectrum. When λ is large, $\left|\mathbf{F}_{:,k}^{\mathrm{H}}\mathbf{C}_{L+1}\mathbf{T}^{(m)}\right|^2$ becomes a closer approximation for $I_{\mathbf{T}}(k)$. Afterwards, if the matrix $\sum_{l=1}^{L}\mathbf{C}_l^{\mathrm{H}}\mathrm{diag}(|\mathbf{R}^{(m-1)}|^2)_{n\times n}\mathbf{C}_l + \lambda\mathbf{C}_{L+1}^{\mathrm{H}}\mathbf{C}_{L+1}$ is invertible, then (17) has a closed solution given by

$$\mathbf{T}^{(m)} = \left(\sum_{l=1}^{L}\mathbf{C}_l^{\mathrm{H}}\mathrm{diag}\left(|\mathbf{R}^{(m)}|^2\right)_{n\times n}\mathbf{C}_l + \lambda\mathbf{C}_{L+1}^{\mathrm{H}}\mathbf{C}_{L+1}\right)^{-1}\left(\sum_{l=1}^{L}\mathbf{C}_l^{\mathrm{H}}\left(\overline{\mathbf{R}^{(m)}}\odot\mathbf{\Psi}_l^{(m)}\right) + \lambda\mathbf{C}_{L+1}^{\mathrm{H}}\mathbf{\Psi}_{L+1}^{(m)}\right).$$

Else, the gradient descent algorithm can also be applied to estimate the solution of (17).

The calculation of the initialization $\mathbf{R}^{(0)}$ and $\mathbf{T}^{(0)}$ for the CB-NPRS algorithm is a two-step process. First, we proceed like in the B-NPRS algorithm and initialize the sample function $\mathbf{R}^{(0)}$ and a preliminary analyzer function $\tilde{\mathbf{T}}^{(0)}$. The correct initialization for the analyzer $\mathbf{T}^{(0)}$ is then achieved in the second step by projecting $\tilde{\mathbf{T}}^{(0)}$ into the set $\{\mathbf{T}||\mathbf{F}_{:,k}^{\mathrm{H}}\mathbf{C}_{L+1}\mathbf{T}|^2 = I_{\mathbf{T}}(k), 1 \le k \le K\}$.

2. Numerical simulations

To test the ability of the CB-NPRS algorithm, we first apply this method to the four setups discussed in the previous sections. As shown in Figs. 26-29, the CB-NPRS method performs well across all four cases, successfully recovering both the intensity and phase of the target and the analyzer. While the CB-NPRS method produces slightly larger measured and relative errors for the target compared to the NPRS method, it requires only the time spectrum of the analyzer.

3. Experimental results

Finally, the CB-NPRS algorithm is tested on the experimental data set presented in the main text for a nuclear forward scattering setup with aligned magnetization. The retrieved spectra are presented in Fig. 30. The quality of the retrieved energy spectra is similar to the ones obtained with the B-NPRS algorithm, retrieving the main features of the energy spectrum with more oscillations of the baseline as compared to the NPRS algorithm. The retrieval of the phase works equally well for NPRS, B-NPRS and CB-NPRS, only missing the very abrupt jumps at approx. $\pm 30\Gamma_0$. Also in this case, the measurement error is

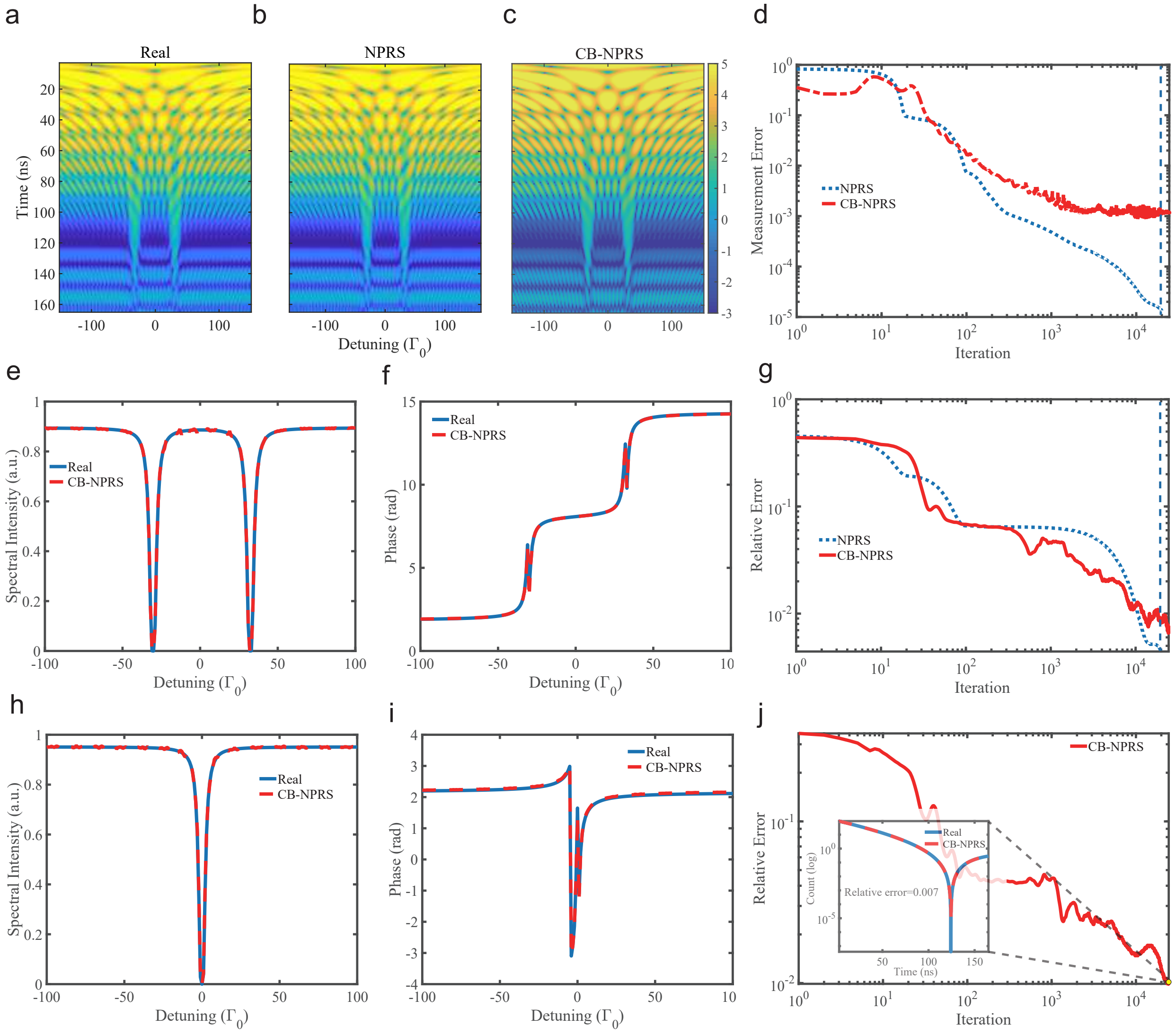

SUPPLEMENTARY FIG. 26. **Numerical results for the α-Fe sample of $2.3\,\mu$m with an aligned magnetization and an analyzer of 1 μm effective thickness.** **a-c** are the real 2D spectrum, and 2D spectrum recovered by the NPRS and CB-NPRS algorithms, respectively. **d** records the measurement error of NPRS and CB-NPRS. **e-f**, and **h-i** show the recovered spectral intensities and phases of the target (**R**) and the analyzer (**T**). **g** and **j** record the relative errors for the target and the analyzer. The reconstructed CB-NPRS time spectrum of the analyzer at the end of the iteration range is shown in the inset of **j**.

slightly smaller than that of NPRS, a phenomenon which is attributed to overfitting of the experimental noise.

In addition, we can use a Fourier transform to calculate retrieved time spectra, which can be compared to time spectra taken in the experiment. The comparison for the sample time spectra is presented in Fig. 30**g**. We find that CB-NPRS performs better than B-NPRS,

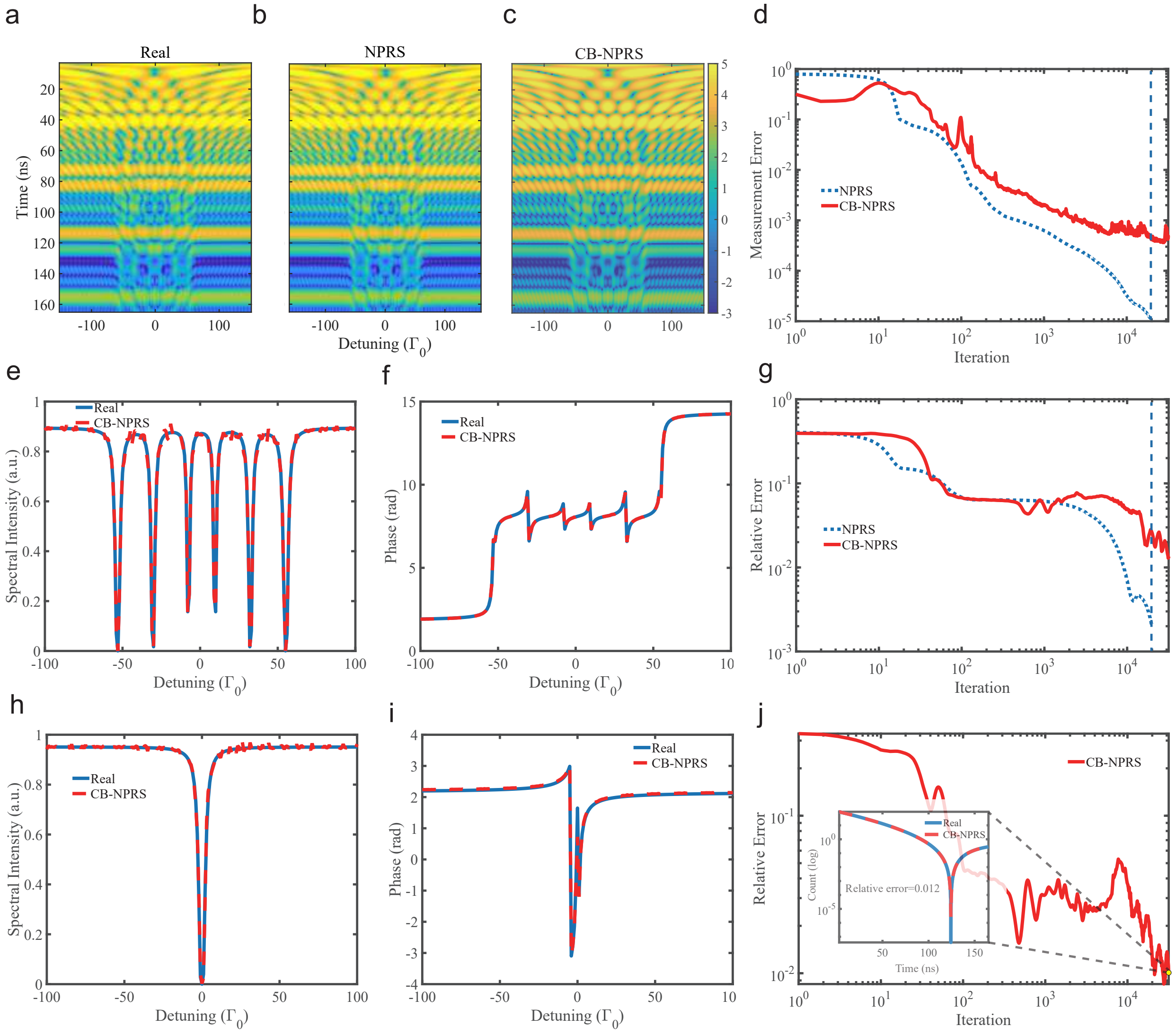

SUPPLEMENTARY FIG. 27. **Numerical results for an α-Fe sample of** $2.3\,\mu$**m with random magnetization.** **a-c** are the real 2D spectrum, and 2D spectrum recovered by the NPRS and CB-NPRS algorithms, respectively. **d** records the measurement error of NPRS and CB-NPRS. **e-f**, and **h-i** show the recovered spectral intensities and phases of the target (**R**) and the analyzer (**T**). **g** and **j** record the relative errors for the target and the analyzer. The reconstructed CB-NPRS time spectrum of the analyzer at the end of the iteration range is shown in the inset of **j**.

but still slightly less good than NPRS. As expected (since it was used as a constraint), the analyzer time spectra in Fig. 30**j** show very good agreement of CB-NPRS to the experimental spectrum.

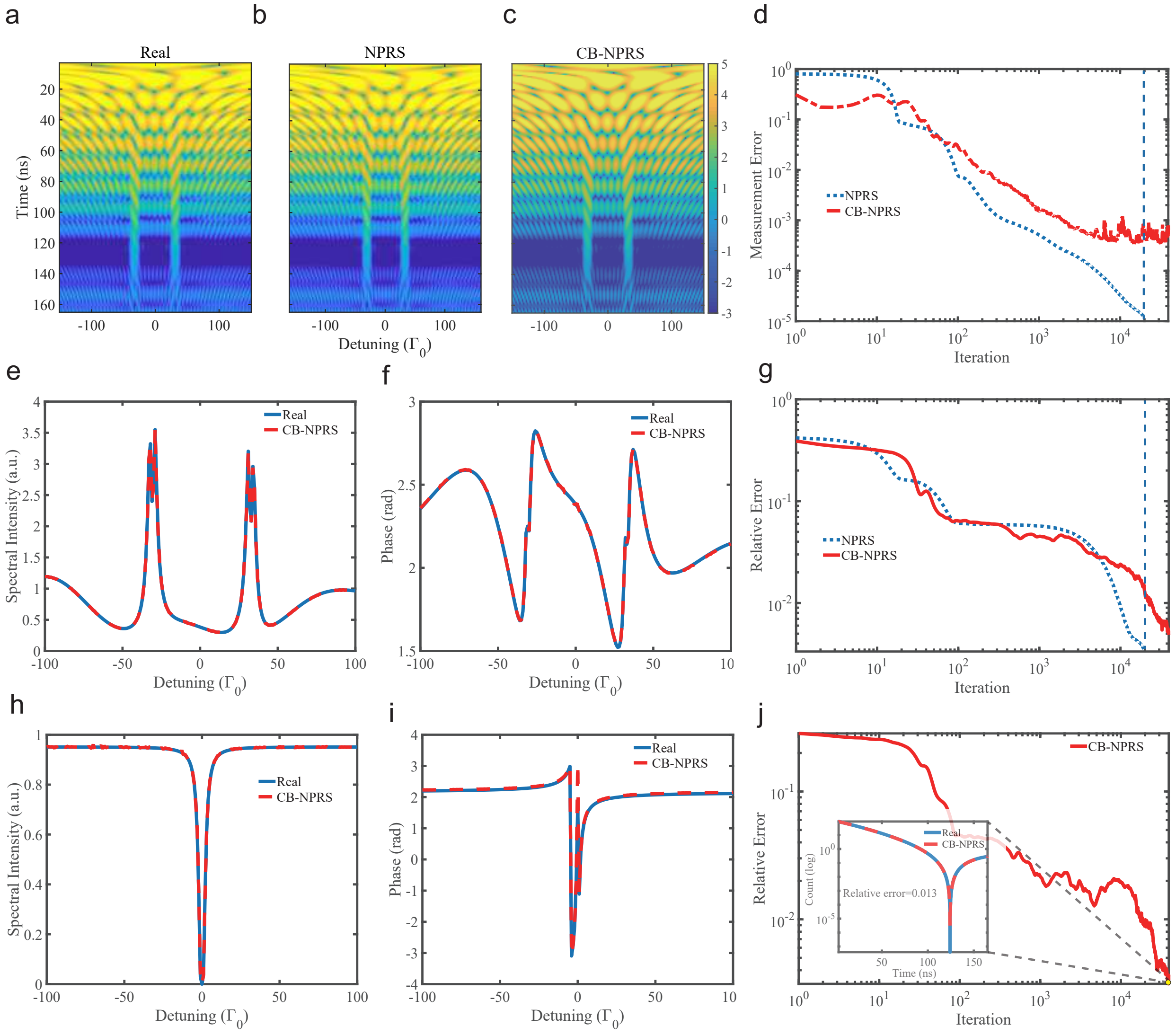

SUPPLEMENTARY FIG. 28. **Numerical results for the setup with fast mechanical motion with the displacement** 0.43 **Å applied on an** α**-Fe sample of** $2.0\,\mu$**m with aligned magnetization.** **a-c** are the real 2D spectrum, and 2D spectrum recovered by the NPRS and CB-NPRS algorithms, respectively. **d** records the measurement error of NPRS and CB-NPRS. **e-f**, and **h-i** show the recovered spectral intensities and phases of the target (**R**) and the analyzer (**T**). **g** and **j** record the relative errors for the target and the analyzer. The reconstructed CB-NPRS time spectrum of the analyzer at the end of the iteration range is shown in the inset of **j**.

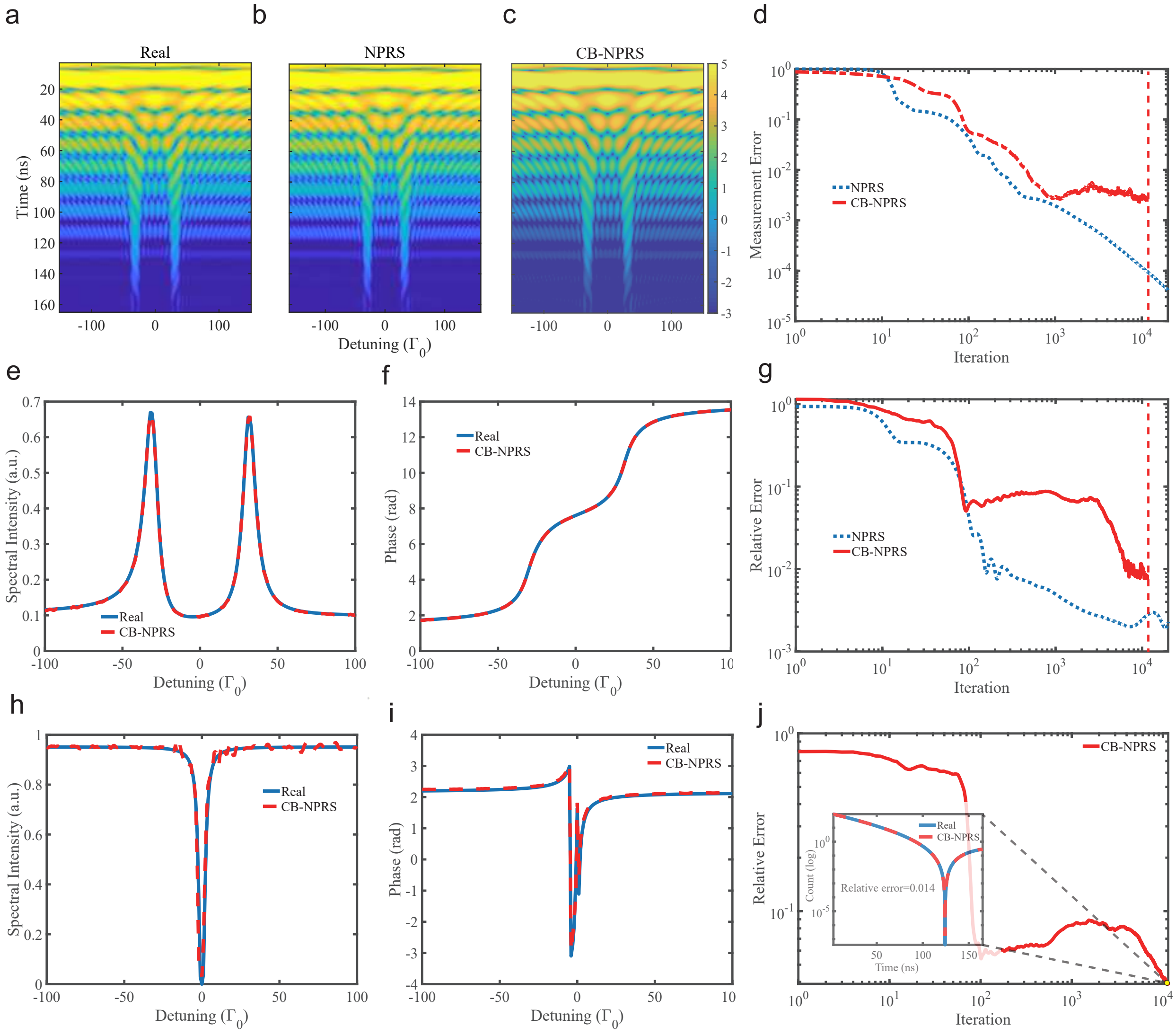

SUPPLEMENTARY FIG. 29. **Numerical results for a thin-film cavity with the structure Ta (2 nm)/B (17 nm)/**^{57}Fe**(3 nm)/B (7 nm)/Ta (9 nm) at the incidence angle** 3.5 **mrad.** **a-c** are the real 2D spectrum, and 2D spectrum recovered by the NPRS and CB-NPRS algorithms, respectively. **d** records the measurement error of NPRS and CB-NPRS. **e-f**, and **h-i** show the recovered spectral intensities and phases of the target (**R**) and the analyzer (**T**). **g** and **j** record the relative errors for the target and the analyzer. The reconstructed CB-NPRS time spectrum of the analyzer at the end of the iteration range is shown in the inset of **j**.

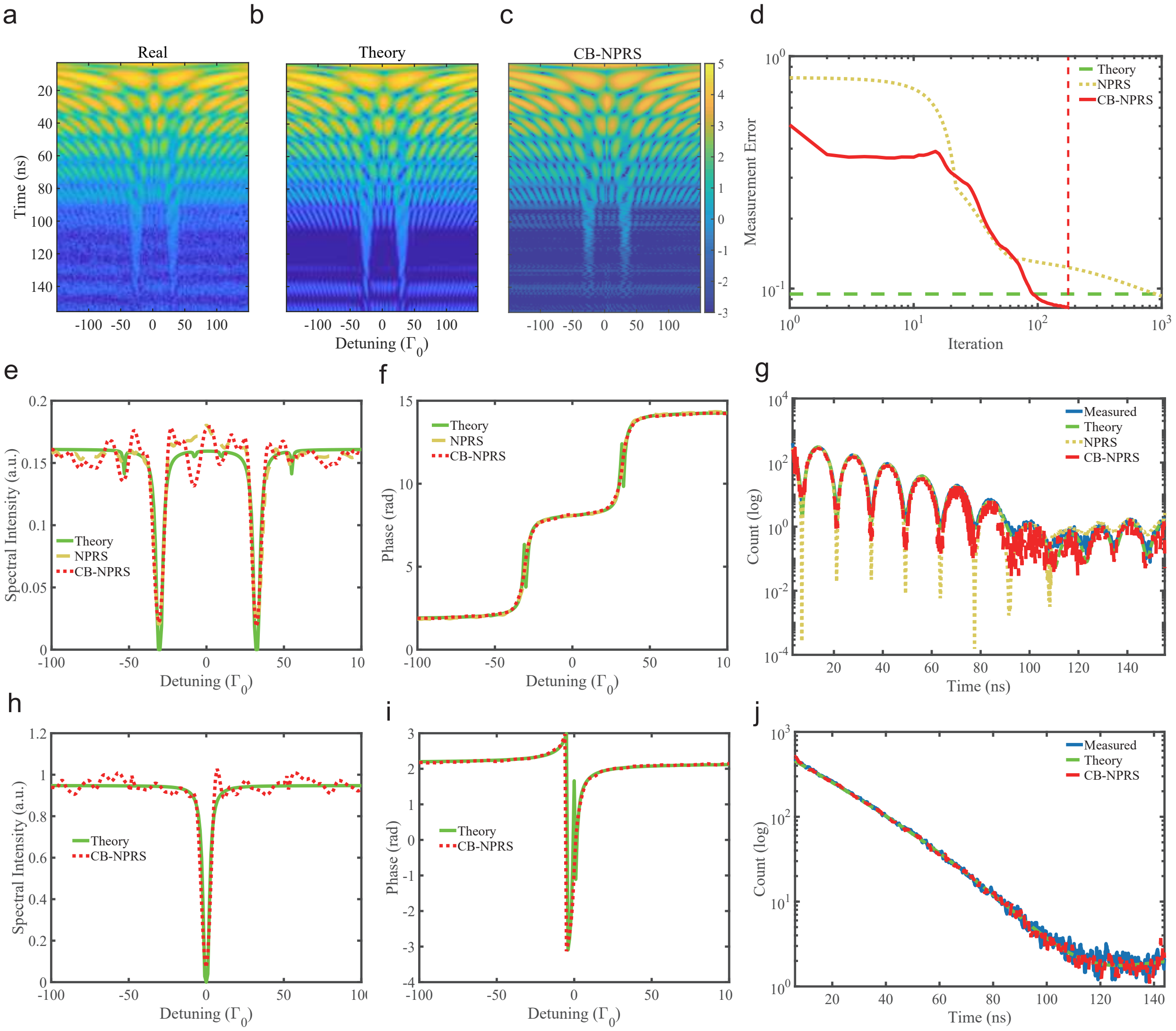

SUPPLEMENTARY FIG. 30. **Experimental results for α-Fe sample of $2.3\,\mu$m with an aligned magnetization and an analyzer of 1 μm effective thickness.** **a-c** are the real 2D spectrum, and 2D spectrum recovered by the NPRS and CB-NPRS algorithms, respectively. **d** records the measurement error of NPRS and CB-NPRS. **e-f**, and **h-i** show the recovered spectral intensities and phases of the target (**R**) and the analyzer (**T**). **g** and **j** plot the recovered and measured time spectra of the target and the analyzer.

SUPPLEMENTARY REFERENCES

[1] Bendory, T., Eldar, Y. C. & Boumal, N. Non-convex phase retrieval from stft measurements. *IEEE Transactions on Information Theory* **64**, 467–484 (2017).

[2] Alaifari, R. & Wellershoff, M. Uniqueness of stft phase retrieval for bandlimited functions. *Applied and Computational Harmonic Analysis* **50**, 34–48 (2021).

[3] Grohs, P. & Rathmair, M. Stable gabor phase retrieval and spectral clustering. *Communications on Pure and Applied Mathematics* **72**, 981–1043 (2019).

[4] Wang, G., Giannakis, G. B. & Eldar, Y. C. Solving systems of random quadratic equations via truncated amplitude flow. *IEEE Transactions on Information Theory* **64**, 773–794 (2016).

[5] Nesterov, Y. A method for solving the convex programming problem with convergence rate $\mathcal{O}(1/k^2)$. *Soviet Mathematics Doklady* **27**, 372–376 (1983).

[6] Heeg, K. P. *et al.* Spectral narrowing of x-ray pulses for precision spectroscopy with nuclear resonances. *Science* **357**, 375–378 (2017).

[7] Heeg, K. P. *et al.* Vacuum-assisted generation and control of atomic coherences at x-ray energies. *Phys. Rev. Lett.* **111**, 073601 (2013).

[8] Heeg, K. P. *et al.* Tunable subluminal propagation of narrow-band x-ray pulses. *Physical Review Letters* **114**, 203601 (2015).

[9] Smirnov, G. Nuclear resonant scattering of synchrotron radiation. *Hyperfine Interactions* **97**, 551–588 (1996).

[10] Sturhahn, W. Conuss and phoenix: Evaluation of nuclear resonant scattering data. *Hyperfine Interactions* **125**, 149—172 (2000).

[11] Yaroslavtsev, S. & Chumakov, A. Synchrotron mössbauer source: trade-off between intensity and linewidth. *J. Synchrotron Radiat.* **29**, 1329 (2022).

[12] Rodenburg, J. M. Ptychography and related diffractive imaging methods. *Advances in imaging and electron physics* **150**, 87–184 (2008).

[13] Rodenburg, J. M. & Faulkner, H. M. A phase retrieval algorithm for shifting illumination. *Applied physics letters* **85**, 4795–4797 (2004).

[14] Bauschke, H. H., Combettes, P. L. & Luke, D. R. Phase retrieval, error reduction algorithm, and fienup variants: a view from convex optimization. *JOSA A* **19**, 1334–1345 (2002).

[15] Wen, Z., Yang, C., Liu, X. & Marchesini, S. Alternating direction methods for classical and

ptychographic phase retrieval. *Inverse Problems* **28**, 115010 (2012).

[16] Lindstrom, S. B. & Sims, B. Survey: sixty years of douglas–rachford. *Journal of the Australian Mathematical Society* **110**, 333–370 (2021).

[17] Röhlsberger, R., Schlage, K., Sahoo, B., Couet, S. & Rüffer, R. Collective lamb shift in single-photon superradiance. *Science* **328**, 1248–1251 (2010).

[18] Hesse, R., Luke, D. R., Sabach, S. & Tam, M. K. Proximal heterogeneous block implicit-explicit method and application to blind ptychographic diffraction imaging. *SIAM Journal on Imaging Sciences* **8**, 426–457 (2015).

[19] Chang, H., Enfedaque, P. & Marchesini, S. Blind ptychographic phase retrieval via convergent alternating direction method of multipliers. *SIAM Journal on Imaging Sciences* **12**, 153–185 (2019).

[20] Bendory, T., Edidin, D. & Eldar, Y. C. Blind phaseless short-time fourier transform recovery. *IEEE Transactions on Information Theory* **66**, 3232–3241 (2019).

[21] Li, J. Solving blind ptychography effectively via linearized alternating direction method of multipliers. *Journal of Scientific Computing* **94**, 19 (2023).

[22] Chang, H., Yang, L. & Marchesini, S. Fast iterative algorithms for blind phase retrieval: A survey. In *Handbook of Mathematical Models and Algorithms in Computer Vision and Imaging: Mathematical Imaging and Vision*, 139–174 (Springer, 2023).

[23] Fannjiang, A. & Chen, P. Blind ptychography: uniqueness and ambiguities. *Inverse Problems* **36**, 045005 (2020).
